# Carbon Dioxide Hydrogenation to Formate Catalyzed by a Bench-Stable, Non-Pincer-Type Mn(I) Alkylcarbonyl Complex

**DOI:** 10.1021/acs.organomet.0c00710

**Published:** 2021-04-20

**Authors:** Sylwia Kostera, Stefan Weber, Maurizio Peruzzini, Luis F. Veiros, Karl Kirchner, Luca Gonsalvi

**Affiliations:** † Consiglio Nazionale delle Ricerche (CNR), Istituto di Chimica dei Composti Organometallici (ICCOM), Via Madonna del Piano 10, 50019 Sesto Fiorentino (Firenze), Italy; ‡ Institute of Applied Synthetic Chemistry, Vienna University of Technology, Getreidemarkt 9/163-AC, A-1060 Vienna, Austria; § Centro de Química Estrutural and Departamento de Engenharia Química, Instituto Superior Técnico, Universidade de Lisboa, Av Rovisco Pais, 1049-001 Lisboa, Portugal

## Abstract

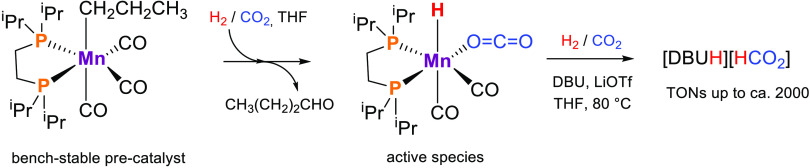

The catalytic reduction of carbon dioxide is a process of growing interest for the use of this simple and abundant molecule as a renewable building block in C1-chemical synthesis and for hydrogen storage. The well-defined, bench-stable alkylcarbonyl Mn(I) bis(phosphine) complex *fac*-[Mn(CH_2_CH_2_CH_3_)(dippe)(CO)_3_] [dippe = 1,2-bis(diisopropylphosphino)ethane] was tested as an efficient and selective non-precious-metal precatalyst for the hydrogenation of CO_2_ to formate under mild conditions (75 bar total pressure, 80 °C), in the presence of a Lewis acid co-catalyst (LiOTf) and a base (DBU). Mechanistic insight into the catalytic reaction is provided by means of density functional theory (DFT) calculations.

## Introduction

In recent years, the increasing concentration of CO_2_ in the atmosphere and its contribution to climate change made decision makers and society at large more aware of the need to curb emissions of this greenhouse gas. As an alternative to simple adsorption and storage, many scientists worldwide have made a case for reuse of CO_2_, as it may represent an abundant, renewable, and cheap feedstock for C1-chemical synthesis.^1^ In brief, two CO_2_ utilization pathways are possible: a nonreductive approach, involving the incorporation of CO_2_ in reactive organic molecules such as epoxides, aziridines, alkenes, etc., and a reductive approach, to obtain simple C1 molecules such as formic acid (HCO_2_H), formaldehyde (HCHO), methanol (CH_3_OH), dimethyl ether (CH_3_OCH_3_), methane (CH_4_), or higher hydrocarbons.^2^ Among these products, methanol and formic acid find large use as bulk chemicals in industrial and laboratory applications and are receiving attention as fuels (MeOH) and as highly promising liquid organic hydrogen carriers (LOHC), to generate H_2_ on demand by dehydrogenation reactions in the presence of suitable homogeneous or heterogeneous catalysts.^3^ In this way, the use of CO_2_ represents an opportunity for the realization of a sustainable, zero-carbon-emission cycle for hydrogen storage and delivery.^4^

Formic acid has a steadily growing market as a bulk chemical, especially in the Asian basin, due to the increasing need in agriculture for silage and as preservant in food. Other traditional applications include its use as a strong acid in wood pulping, leather, and textile industries. Formates have also important applications, for example, as auxiliary agents in leather treatment, for deicing at airports, in electroplating and photographic fixing baths, and in constructions as an additive to concrete.^5^ HCO_2_H is currently obtained industrially from the hydrolysis of HCO_2_Me, in turn derived from fossil feedstock as one of the products of methanol carbonylation. A sustainable alternative using renewable, non-fossil-based feedstocks is therefore highly desirable. HCO_2_H can indeed be obtained from the 100% atom-efficient reaction between CO_2_ and H_2_ under different conditions of temperature and total pressure, providing that key issues are solved. The first major hurdle in CO_2_ hydrogenation is the endergonic character of the reaction due to the large entropic contribution (Δ*S*^0^ = −215 kJ mol^–1^); however, the reaction can be made exoergonic in the presence of strong bases or using highly polar solvents such as water.^6^ Second, CO_2_ is a rather chemically inert molecule; thus, efficient catalysts are needed to overcome activation barriers and operate the process under mild conditions. Homogeneous catalysts, based on tailored organometallic or coordination complexes, were studied over the years by different research groups worldwide, showing that by fine tuning of the ancillary ligands stabilizing the metal center, high activities and selectivities could be achieved under relatively mild reaction conditions.^4,6^

Both noble- and base-metal complexes were shown to be able to catalyze CO_2_ hydrogenation to formate. The state-of-the-art for noble-metal-catalyzed processes is held by Nozaki and co-workers with the use of the pincer-type tris(hydrido) complex [Ir(H)_3_(PNP-*i*Pr)] as a catalyst [PNP-*i*Pr = 2,6-bis((diisopropylphosphanyl)methyl)pyridine], reaching outstanding TON = 3 500 000 and TOF = 73 000 h^–1^ with KOH as a base in tetrahydrofuran (THF), 60 bar H_2_/CO_2_ (1:1), 120 °C, 48 h.^7^ In the case of earth-abundant metals, in recent years, the attention has been focused principally on Fe,^8^ although interesting results were reported also with Co,^9^ Ni,^10^ and Cu.^11^ Very recently, Klankermayer and co-workers established the new state-of-the-art for 3d metal-catalyzed CO_2_ hydrogenation with the system obtained *in situ* by the combination of Ni(BF_4_)_2_·6H_2_O (0.002 μmol) and the tetradentate ligand tris-[2-(diphenylphosphino)ethyl]amine (NP_3_, 1 equiv to Ni) in CH_3_CN.^12^ In the presence of DBU as a base, 90 bar H_2_/CO_2_ (2:1), 120 °C, 72 h, unsurpassed TON = 4 650 710 and TOF = 64 593 h^–1^ were achieved, showing that earth-abundant metals can efficiently compete with noble-metal counterparts.

**Chart 1:**
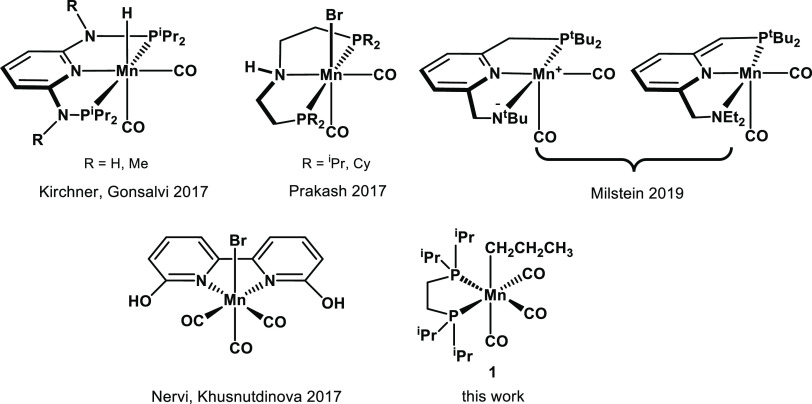
Mn(I) Pincer-Type (Top)^18,19,21^ and Non-Pincer-Type Complexes (Bottom)^22^ Used as Catalysts or Precatalysts for CO_2_ Hydrogenation

Since 2016 manganese, the third most abundant metal in the Earth’s crust after Fe and Ti has witnessed a true renaissance for use in homogeneous catalysis, including dehydrogenation,^13^ hydrogenation,^14^ alcohol β-methylation,^15^ aminomethylation reactions,^16^ etc. These and other applications have been highlighted in recent review articles.^17^ Only a few examples of Mn-catalyzed CO_2_ hydrogenation have appeared so far in the literature, mainly involving pincer-type complexes (Chart 1, top). We jointly reported the first example of Mn(I)-catalyzed hydrogenation of CO_2_ to formate in the presence of the hydridocarbonyl complex [MnH(PNP^NH^-*i*Pr)(CO)_2_]. At catalyst loadings as low as 0.002 mol %, TONs up to 10 000 and quantitative yields of formate were obtained after 24 h using DBU as a base, 80 bar H_2_/CO_2_ (1:1) at 80 °C. Remarkably, TONs higher than 30 000 could be achieved adding LiOTf as a co-catalyst.^18^ Prakash and co-workers showed the use of complex [MnBr(^R^PNP)(CO)_2_] [^R^PNP = bis(2-(dialkylphosphino)ethyl)amine; R = *i*Pr, Cy] in the one-pot CO_2_ hydrogenation to CH_3_OH in the presence of amines. The first step of the sequential reaction was proposed to be the two-electron reduction of CO_2_ to formate, which reacts with the amine to give an intermediate formamide. This is in turn reduced to CH_3_OH, giving back the initial amine.^19^ In the same year, Pathak and co-workers highlighted mechanistic details on base-free CO_2_ hydrogenation with similar PNP-type Mn complexes by density functional theory (DFT) calculations.^20^ Milstein and co-workers reported the use of Mn(I) complexes with PNN pincer ligands, able to activate CO_2_ in different modes. Under catalytic conditions, namely, 10 mol % of catalyst in THF, KOH as a base, 60 bar H_2_/CO_2_ (1:1), 110 °C, 60 h, up to 23% yield of HCO_2_K was obtained.^21^ Nervi, Khusnutdinova, and co-workers published the so far only example of non-pincer-type Mn(I) catalysts for CO_2_ hydrogenation, stabilized by functionalized bipyridyl-type ligands (Chart 1, bottom). It was shown that with *o*-OH-substituted complexes (0.015 mol %) as catalysts in CH_3_CN, DBU as a base, 60 bar H_2_/CO_2_ (1:1), 65 °C, formate was obtained in 98% yield after 24 h, reaching a maximum TON of 6250.^22^

Very recently, it was shown that long-known Mn(I) complexes stabilized by chelating bis(phosphines) such as 1,2-bis(di-*i*-propylphosphino)ethane (dippe) could be used as efficient catalysts for alkene,^23^ ketone, and nitrile hydrogenation.^24^ Inspired by these results, we were interested to study the properties of the bench-stable alkylcarbonyl Mn(I) complex *fac*-[Mn(CH_2_CH_2_CH_3_)(dippe)(CO)_3_] (**1**) shown in Chart 1 (bottom) as a precatalyst for the homogeneous CO_2_ hydrogenation to formate. The results of the catalytic tests, including a screening of the reaction conditions and the effect of a Lewis acid co-catalyst, are hereby presented.

## Results and Discussion

**Scheme 1:**
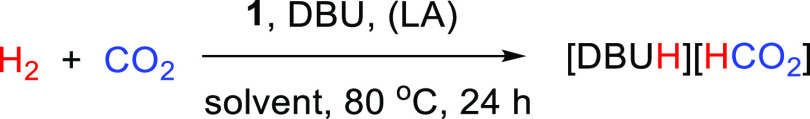
CO_2_ Hydrogenation to Formate in the Presence of Precatalyst **1** and DBU, with Possible Addition of a Lewis Acid (LA) Co-catalyst

Initially, CO_2_ hydrogenation (Scheme 1) was tested using **1** under the conditions previously applied^18^ with [MnH(PNP^NH^-*i*Pr)(CO)_2_], *i.e*., in the presence of 1,8-diazabicycloundec-7-ene (DBU) as a base, 80 °C, under H_2_/CO_2_ (1:1) 60 bar total pressure, using either a THF/H_2_O (10/1) solvent mixture or EtOH. After 24 h, no conversion was observed in either solvents. The dark brown color of the solutions and the presence of a dark precipitate at the end of the tests indicate that the activated form of **1** (*vide infra*) decomposes in these solvents under catalytic conditions.

By changing the solvent to dry THF, no catalyst decomposition was observed and substrate conversion was noted at the end of the reactions. The results of the first screening on the effects of different catalyst-to-base ratios and total gas pressure are reported in Table 1.

**Table 1:** Catalytic CO_2_ Hydrogenation with 1 Using a H_2_/CO_2_ = 1:1 Gas Mixture^a^

entry	**1**/DBU	pH_2_/pCO_2_ (bar)	time (h)	TON^b^	yield (%)^c^
1	1/1000	30/30	24	377	37.5
2	1/1000	20/20	24	198	19.7
3	1/1000	30/30	48	425	42.3
4	1/1000	30/30	72	568	56.5
5	1/5000	30/30	24	1077	21.4
6	1/10 000	30/30	24	156	1.5
7	1/50 000	30/30	24	235	0.5
8	1/10 000	40/40	24	404	4.0

a Reaction conditions: catalyst **1**, 0.2–10 μmol; DBU, 10 mmol; THF, 5.5 mL; H_2_/CO_2_ (1:1) pressure; 80 °C.

b TON = (mmol formate)/(mmol catalyst).

c Yield = [(mmol formate)/(mmol DBU)] × 100. The amount of formate was calculated from the integration of the corresponding ^1^H NMR signal in D_2_O against an internal standard (DMF). All experiments were repeated at least twice to check for reproducibility; average error, ca. 6%.

Using a **1**/DBU ratio of 1:1000, formate was obtained in 37.5% yield with respect to DBU, with TON = 377 (entry 1). The total pressure was then decreased to 40 bar, but as expected, this caused a drop in yield and TON (entry 2). Under the standard 60 bar total pressure, an increase in productivity was achieved by running the tests for longer times, namely, 48 and 72 h (entries 3 and 4, respectively), reaching the highest yield (56.5%) and TON of 568 under these conditions (entry 4). Next, the amount of catalyst was decreased to **1**/DBU ratios of 1:5000, 1:10 000, and 1:50 000 (entries 5, 6, and 7, respectively), running the tests at 60 bar, 80 °C, 24 h. At an optimal 1:5000 ratio, TON increased to 1077; however, yield decreased to 21.4%. Lower **1**/DBU ratios led to poor results. A slight improvement was possible at **1**/DBU = 1:10 000 by increasing the total gas pressure to 80 bar (entry 8).

The next optimization step was to study the effect of higher H_2_/CO_2_ ratios on the catalytic activity. Indeed, in the case of alkene hydrogenation with **1**, it was previously demonstrated that catalyst activation occurred under a H_2_ pressure of 50 bar.^22^ The results are summarized in Table 2.

**Table 2:** Catalytic CO_2_ Hydrogenation with 1 Using Different H_2_/CO_2_ Partial Pressure Ratios^a^

entry	**1**/DBU	pH_2_/pCO_2_ (bar)	TON^b^	yield (%)^c^
1	1/1000	50/25	1000	100
2	1/1000	60/20	1000	100
3	1/2000	50/25	540	26.8
4	1/5000	50/25	98	1.9
5	1/10 000	50/25	109	1.1

a Reaction conditions: catalyst **1**, 1–10 μmol; DBU, 10 mmol; THF, 5.5 mL; H_2_/CO_2_ (2:1 or 3:1) pressure; 80 °C, 24 h.

b TON = (mmol formate)/(mmol catalyst).

c Yield = [(mmol formate)/(mmol DBU)] × 100. The amount of formate was calculated from the integration of the corresponding ^1^H NMR signal in D_2_O against an internal standard (DMF). All experiments were repeated at least twice to check for reproducibility; average error, ca. 6%.

To our delight, the change of gas mixture ratio improved the catalytic performance, and both 2:1 and 3:1 H_2_/CO_2_ ratios gave quantitative yields in formate using a **1**/DBU ratio of 1:1000 (entries 1 and 2). In an attempt to increase further the TON values, lower catalyst loadings were used (entries 3–5) using a H_2_/CO_2_ = 2:1 ratio, but in this case, a notable drop in activity was observed.

Next, the effect of a Lewis acid (LA) addition as a co-catalyst was tested. The effect of LAs in favoring accessible transition states in CO_2_ hydrogenation reaction pathways has been demonstrated in detail, especially in combination with pincer-type complexes of base metals.^25^ In keeping with our previously published results obtained with Mn(I) pincer-type catalysts,^18^ LiOTf was chosen as a suitable LA to promote CO_2_ hydrogenation to formate, using 75 bar total pressure at a 2:1 H_2_/CO_2_ gas mixture and a 1:2000 ratio of **1**/DBU. The results are reported in Table 3.

**Table 3:** Catalytic CO_2_ Hydrogenation with 1, Screening of the Effect of Lewis Acid (LA) Co-catalyst under Various Conditions^a^

entry	**1**/DBU	**1**/LA	LA/DBU	TON^b^	yield (%)^c^
1	1/2000	1/100	0.05	1104	54.8
2^d^	1/2000	1/100	0.05	1988	98.7
3^e^	1/2000	1/100	0.05	85	4.2
4	1/2000	1/200	0.1	135	6.4
5	1/2000	1/50	0.025	678	33.7
6	1/5000	1/250	0.05	238	4.7

a Reaction conditions: catalyst **1**, 2–5 μmol; DBU, 10 mmol; LA = LiOTf, 0.25–1.0 mmol; THF, 5.5 mL; H_2_/CO_2_ (2:1), 75 bar total pressure; 80 °C, 24 h.

b TON = (mmol formate)/(mmol catalyst).

c Yield = [(mmol formate)/(mmol DBU)] × 100. The amount of formate was calculated from the integration of the corresponding ^1^H NMR signal in D_2_O against an internal standard (DMF).

d As above, 48 h.

e As above, 100 °C, 24 h. All experiments were repeated at least twice to check for reproducibility; average error, ca. 6%.

**Scheme 2:**
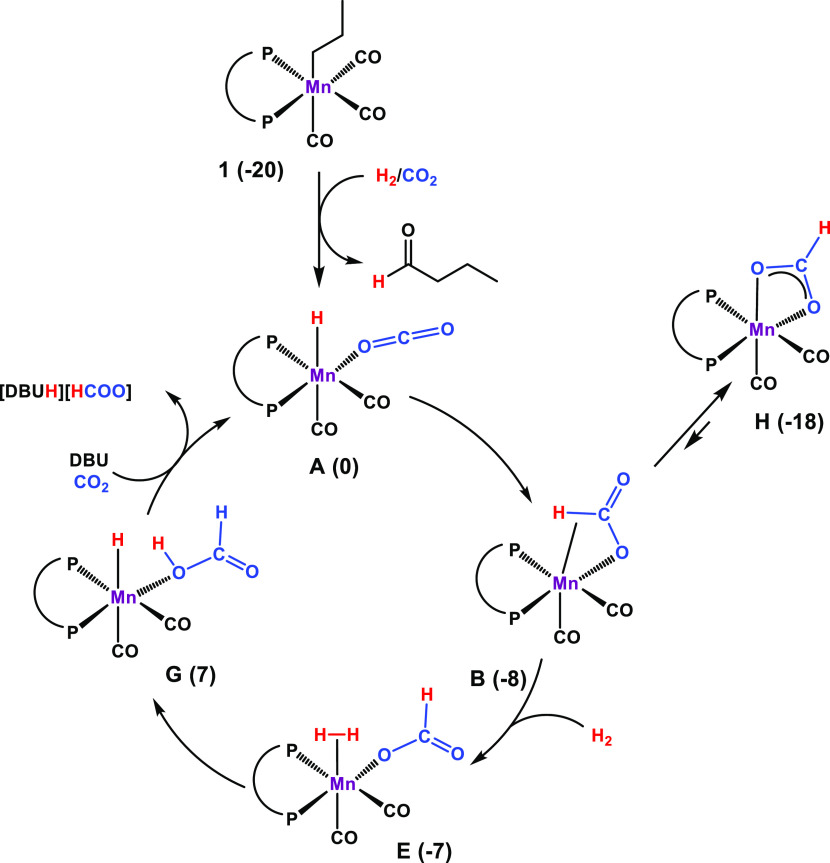
Proposed Catalytic Cycle for the Hydrogenation of CO_2_ to Formate Starting from **1** in the Presence of DBU DFT calculated free energy values (kcal/mol) in parentheses.

In the presence of added LiOTf (0.5 mmol, **1**/LiOTf = 1:100), formate was obtained in a 54.8% yield (TON = 1104, entry 1) after 24 h. At a longer reaction time (48 h, entry 2), yields up to 98.7% were observed, corresponding to a TON of 1988. The effect of the temperature was tested by increasing it from 80 to 100 °C on a 24 h run, but this resulted in a drop of activity (4.2% yield, entry 3), likely due to the poor catalyst stability at this temperature. Increasing the LiOTf amount to 1.0 mmol (**1**/LiOTf = 1:200), at 80 °C for 24 h, caused a decrease in TON (entry 4). As previously suggested, such an effect may be attributed to the limited LiOTf solubility in such a solvent mixture.^8c^ On the other hand, using 0.25 mmol of LiOTf (**1**/LiOTf = 1:50) gave a slightly decreased TON = 678 (entry 5) after 24 h compared to the results obtained with 0.5 mmol (entry 1). Based on the results of the catalytic tests and previous studies on **1** as an alkene hydrogenation catalyst,^23^ a simplified mechanism based on DFT calculations is proposed and shown in Scheme 2.

**Figure 1:**
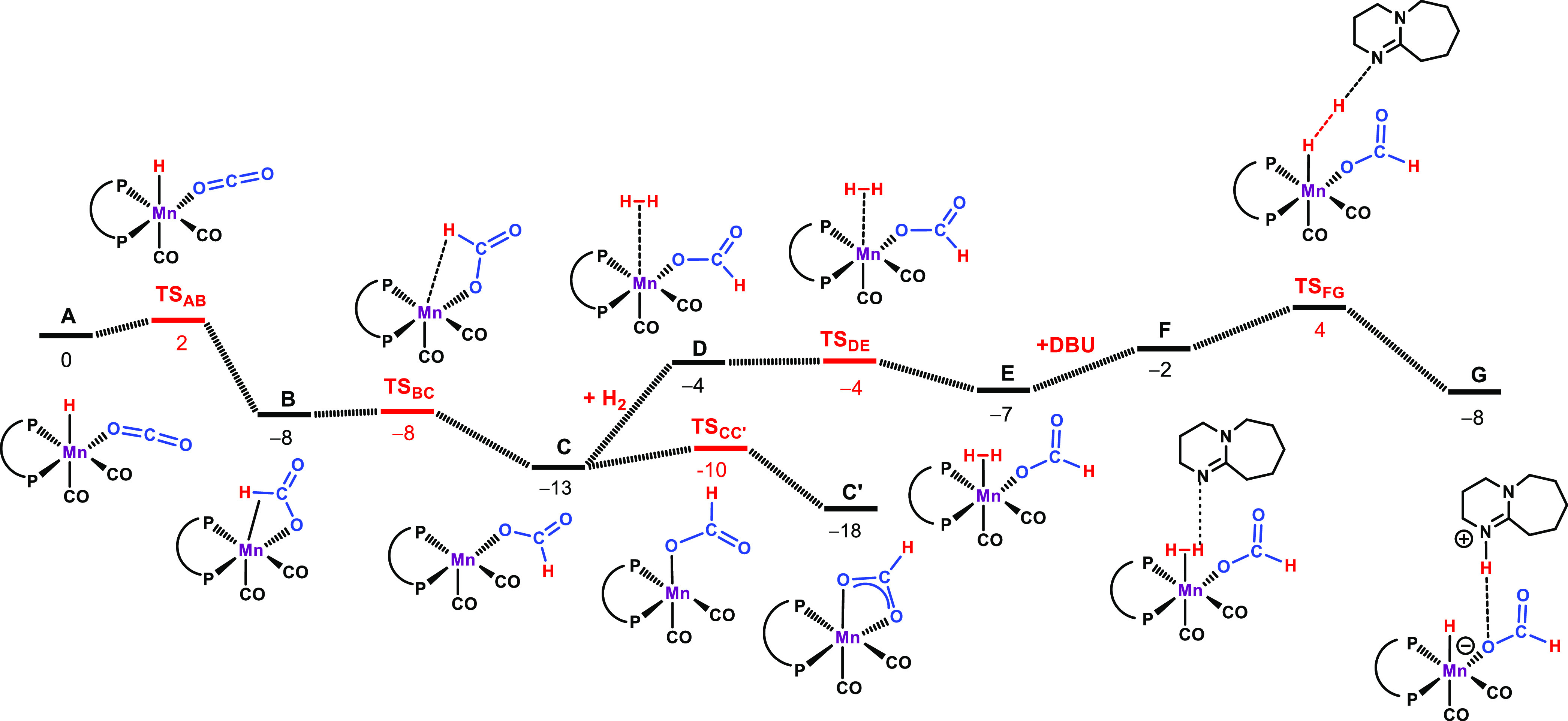
Free energy profile for the formation of formic acid. Free energies (kcal/mol) are referred to [MnH(dippe)(κ^1^-*O*-CO_2_)] (A in the Calculations).

**Scheme 3:**
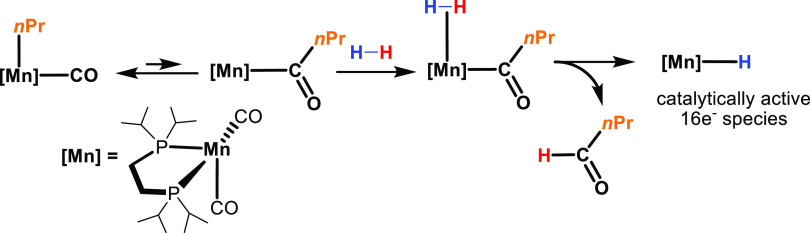
Formation of the 16e^–^ Hydride Intermediate [MnH(dippe)(CO)_2_] upon Reaction of **1** with H_2_

The κ^1^-*O*-CO_2_ hydride complex *cis*-[MnH(dippe)(CO)_2_(κ^1^-*O*-CO_2_)] (**A**) has been chosen as a reference point. The free energy profile calculated for the catalytic reaction is depicted in Figures 1 and S20 (Supporting Information). As already reported recently,^23,24^ precatalyst **1** is initially activated under a pressure of H_2_ to form the highly reactive 16e^–^ hydride intermediate [MnH(dippe)(CO)_2_] by migratory insertion of the CH_2_CH_2_CH_3_ ligand in the Mn–CO bond as shown in Scheme 3. This step is accompanied by the release of 1-butanal, which under these conditions is hydrogenated to butanol as detected by ^1^H NMR spectroscopy. This key activation step is a long-known textbook reaction, demonstrated for this class of complexes as early as in the 1950s and studied further by different authors in following years, and it makes this class of alkyl complexes attractive as bench-stable precursors to sensitive metal hydrido catalysts for hydrogenation reactions.^26^

In the presence of CO_2_, [MnH(dippe)(CO)_2_] is converted into *cis*-[MnH(dippe)(CO)_2_(κ^1^-*O*-CO_2_)] (**A**). The C···H separation is 3.52 Å. Upon rotation of the CO_2_ ligand by ca. 20° around the Mn–O bond, insertion into the Mn–H bond affords the κ^2^-*CH,O*-formato complex **B** through an early transition state (**TS**_**AB**_, Δ*G*^‡^ = 2 kcal/mol) with a long C···H separation (3.27 Å). **B** reacts through a barrierless step to the coordinatively unsaturated species *cis*-[Mn(κ^1^-*O-*OCOH)(dippe)(CO)_2_] (**C**), more stable than **B** by 5 kcal/mol. From **C**, formate rearrangement yields the κ^2^-*O,O*-formate species *cis*-[Mn(κ^2^-*O,O*-OCHO) (dippe)(CO)_2_] (**C′**) that is a dead end in the path and can be viewed as a resting state of the catalyst. This is a very easy process with a barrier of merely 3 kcal/mol and Δ*G* = −5 kcal/mol. The catalytic cycle proceeds from **C** through a parallel path, with addition of a H_2_ molecule to give the dihydrogen complex **E**, which is formed through a 9 kcal/mol barrier. Coordinated H_2_ in intermediate **E** is activated by the base (DBU), giving a formate complex H-bonded to the protonated base (DBUH^+^) in species **G**. The corresponding transition state (**TS**_**FG**_) is a less stable one of the entire path, generating an overall barrier of Δ*G*^‡^ = 22 kcal/mol for the catalytic reaction, measured from the most stable intermediate, the resting state **C′**. The catalytic cycle closes, from **G** back to **A**, with release of the pair [DBUH][HCO_2_] and coordination of a fresh CO_2_ molecule with an associated balance of Δ*G* = 10 kcal/mol. For this system, the LA effect should be to disfavor the isomerization of **B** to **C′** formed as off-cycle species and stabilized by chelate effect. This in turn makes the following hydrogen activation step less energetically demanding, involving the more loosely κ^2^-*CH,O*-bound formate rather than the κ^2^-*O,O*-bound isomer **C′**. Alternatively, in the presence of H_2_, the 16e^–^ active species [MnH(dippe)(CO)_2_] can readily be converted into the dihydrogen hydride species *cis*-[MnH(η^2^-H_2_)(dippe)(CO)_2_] (**H**). In fact, such a complex is more stable than **A** by 12 kcal/mol. On the other hand, this renders the hydride ligand less basic than in **A** and, overall, makes CO_2_ insertion *via* an outer-sphere pathway less favorable. The energy profile for a possible outer-sphere pathway involving **H** is provided in the Supporting Information (Figure S21).

## Conclusions

In summary, we have hereby reported the first example of use of a non-pincer, bis(phosphine)-Mn(I) chelate alkylcarbonyl complex as a precatalyst for CO_2_ hydrogenation to formate under mild reaction conditions (80 °C, 75 bar H_2_/CO_2_) in the presence of an added base (DBU) and a Lewis acid (LiOTf). Although the highest TON was lower than that obtained with our previous system based on the 2,6-bis(aminopyridinyl)diphosphine scaffold, the present study shows that even this class of textbook Mn(I) organometallic complexes can find application in this challenging reaction. The main advantage is the possibility to use a bench-stable alkyl precatalyst to generate *in situ* the active hydrido species under a pressure of hydrogen, and to use a widely available chelating bis(phosphine) ligand to stabilize the metal center. DFT calculations showed that the highest barrier in the reaction pathway (Δ*G*^‡^ = 22 kcal/mol) belongs to the activation of coordinated H_2_ by means of base (DBU), relative to the κ^2^-*O,O*-formate intermediate, the most stable species along the path and a catalyst resting state. A further interesting aspect of this study is the fact that this reaction apparently proceeds *via* an inner-sphere mechanism with the coordinatively unsaturated hydride complex [MnH(dippe)(CO)_2_] as a key intermediate. This species is able to coordinate and insert CO_2_ into the Mn–H bond, thereby initiating the catalytic cycle. It has to be noted that all Mn(I)-catalyzed hydrogenation reactions utilizing dihydrogen as a reducing agent described so far in the literature proceed *via* an outer-sphere pathway where metal–ligand cooperation is essential for dihydrogen activation and cleavage.^17c–17f,20^

## Experimental Section

### General Procedure for Carbon Dioxide Catalytic Hydrogenation

In a typical experiment, the catalytic mixture containing solvent, catalyst, base, and additive (if any) was prepared in a Schlenk tube under nitrogen and subsequently injected into a 40 mL magnetically stirred Teflon-lined stainless steel autoclave built at CNR-ICCOM, kept under a nitrogen atmosphere. Then, the autoclave was pressurized with a H_2_/CO_2_ gas mixture at the desired pressure and placed in an oil bath preheated to the desired temperature under stirring at 500 rpm for the set reaction time. After the run, the autoclave was cooled to <10 °C using an ice bath, the pressure was gently released, and the reaction mixture was transferred to a round-bottom flask. The autoclave beaker was thoroughly rinsed with H_2_O, and the washings were added to the rest of the mixture. The volume of the mixture was then gently reduced using a rotary evaporator at room temperature until a homogeneous mixture was obtained. DMF (300 μL) was added to the mixture as internal standard, and the formate content was determined by integration of the corresponding ^1^H NMR signal vs DMF. D_2_O (ca. 0.7 mL) was added as a deuterated solvent. All tests were repeated at least twice to check for reproducibility.

## Supporting Information Available

General methods and materials; selected NMR spectra; DFT calculations methods (PDF)
xyz coordinates file (xyz)

## Supplementary Material

om0c00710_si_001.pdf

om0c00710_si_002.xyz

## Dedication

Dedicated to Prof. Christian Bruneau for his outstanding contribution to catalysis.

## References

[ref1] aNocito F.; Dibenedetto A. Atmospheric CO_2_ Mitigation Technologies: Carbon Capture Utilization and Storage. Curr. Opin. Green Sustainable Chem. 2020, 21, 34–43. 10.1016/j.cogsc.2019.10.002.

[ref2] aChemical Transformations of Carbon Dioxide. In Topics in Current Chemistry Collections, 1st ed.; Wu X.-F.; Beller M., Eds.; Springer International Publishing, 2018.

[ref3] aBahari N. A.; Isahak W. N. R. W.; Masdar M. S.; Yaakob Z. Clean Hydrogen Generation and Storage Strategies via CO_2_ Utilization into Chemicals and Fuels: a Review. Int. J. Energy Res. 2019, 43, 5128–5150. 10.1002/er.4498.

[ref4] aKlankermayer J.; Wesselbaum S.; Beydoun K.; Leitner W. Selective Catalytic Synthesis Using the Combination of Carbon Dioxide and Hydrogen: Catalytic Chess at the Interface of Energy and Chemistry. Angew. Chem., Int. Ed. 2016, 55, 7296–7343. 10.1002/anie.201507458. 27237963

[ref5] Hietala J.; Vuori A.; Johnsson P.; Pollari I.; Reutemann W.; Kieczka H. Formic Acid. In Ullmann’s Encyclopedia of Industrial Chemistry; Wiley-VCH: Weinheim, Germany, 2000.

[ref6] aJessop P. G.; Joo F.; Tai C.-C. Recent advances in the homogeneous hydrogenation of carbon dioxide. Coord. Chem. Rev. 2004, 248, 2425–2442. 10.1016/j.ccr.2004.05.019.

[ref7] Tanaka R.; Yamashita M.; Nozaki K. Catalytic Hydrogenation of Carbon Dioxide Using Ir(III)-Pincer Complexes. J. Am. Chem. Soc. 2009, 131, 14168–14169. 10.1021/ja903574e. 19775157

[ref8] aCoufourier S.; Gaillard S.; Clet G.; Serre C.; Daturi M.; Renaud J.-L. A MOF-assisted phosphine free bifunctional iron complex for the hydrogenation of carbon dioxide, sodium bicarbonate and carbonate to formate. Chem. Commun. 2019, 55, 4977–4980. 10.1039/C8CC09771B. 30968078

[ref9] aBurgess S. A.; Grubel K.; Appel A. M.; Wiedner E. S.; Linehan J. C. Hydrogenation of CO_2_ at Room Temperature and Low Pressure with a Cobalt Tetraphosphine Catalyst. Inorg. Chem. 2017, 56, 8580–8589. 10.1021/acs.inorgchem.7b01391. 28657717

[ref10] aSivanesan D.; Song K. H.; Jeong S. K.; Kim H. J. Hydrogenation of CO_2_ to Formate Using a Tripodal-Based Nickel Catalyst Under Basic Conditions. Catal. Commun. 2019, 120, 66–71. 10.1016/j.catcom.2018.11.016.

[ref11] aRomero E. A.; Zhao T.; Nakano R.; Hu X.; Wu Y.; Jazzar R.; Bertrand G. Tandem Copper Hydride–Lewis Pair Catalysed Reduction of Carbon Dioxide into Formate with Dihydrogen. Nat. Catal. 2018, 1, 743–747. 10.1038/s41929-018-0140-3.

[ref12] Schieweck B. G.; Westhues N. F.; Klankermayer J. A Highly Active Non-Precious Transition Metal Catalyst for the Hydrogenation of Carbon Dioxide to Formates. Chem. Sci. 2019, 10, 6519–6523. 10.1039/C8SC05230A. 31341604 PMC6611062

[ref13] aLéval A.; Junge H.; Beller M. Manganese(I) κ2-NN Complex-Catalyzed Formic Acid Dehydrogenation. Catal. Sci. Technol. 2020, 10, 3931–3937. 10.1039/D0CY00769B.

[ref14] aDas U. K.; Janes T.; Kumar A.; Milstein D. Manganese Catalyzed Selective Hydrogenation of Cyclic Imides to Diols and Amines. Green Chem. 2020, 22, 3079–3082. 10.1039/D0GC00570C.

[ref15] Schlagbauer M.; Kallmeier F.; Irrgang T.; Kempe R. Manganese-Catalyzed β-Methylation of Alcohols by Methanol. Angew. Chem., Int. Ed. 2020, 59, 1485–1490. 10.1002/anie.201912055. PMC7003965 31743576

[ref16] Mastalir M.; Pittenauer E.; Allmaier G.; Kirchner K. Manganese-Catalyzed Aminomethylation of Aromatic Compounds with Methanol as a Sustainable C1 Building Block. J. Am. Chem. Soc. 2017, 139, 8812–8815. 10.1021/jacs.7b05253. 28628321

[ref17] aReed-Berendt B. G.; Polidano K.; Morrill L. C. Recent Advances in Homogeneous Borrowing Hydrogen Catalysis using Earth-abundant First Row Transition Metals. Org. Biomol. Chem. 2019, 17, 1595–1607. 10.1039/C8OB01895B. 30222171

[ref18] Bertini F.; Glatz M.; Gorgas N.; Stöger B.; Peruzzini M.; Veiros L. F.; Kirchner K.; Gonsalvi L. Carbon Dioxide Hydrogenation Catalysed by Well-Defined Mn (I) PNP Pincer Hydride Complexes. Chem. Sci. 2017, 8, 5024–5029. 10.1039/C7SC00209B. 28970889 PMC5613213

[ref19] Kar S.; Goeppert A.; Kothandaraman J.; Prakash G. K. S. Manganese-Catalyzed Sequential Hydrogenation of CO2 to Methanol via Formamide. ACS Catal. 2017, 7, 6347–6351. 10.1021/acscatal.7b02066.

[ref20] Rawat K. S.; Pathak B. Aliphatic Mn–PNP complexes for the CO_2_ hydrogenation reaction: a base free mechanism. Catal. Sci. Technol. 2017, 7, 3234–3242. 10.1039/C7CY00737J.

[ref21] Kumar A.; Daw P.; Espinosa-Jalapa N. A.; Leitus G.; Shimon L. J. W.; Ben-David Y.; Milstein D. CO_2_ Activation by Manganese Pincer Complexes Through Different Modes of Metal–Ligand Cooperation. Dalton Trans. 2019, 48, 14580–14584. 10.1039/C9DT03088C. 31517365

[ref22] Dubey A.; Nencini L.; Fayzullin R. R.; Nervi C.; Khusnutdinova J. R. Bio-Inspired Mn(I) Complexes for the Hydrogenation of CO_2_ to Formate and Formamide. ACS Catal. 2017, 7, 3864–3868. 10.1021/acscatal.7b00943.

[ref23] Weber S.; Stöger B.; Veiros L. F.; Kirchner K. Rethinking Basic Concepts - Hydrogenation of Alkenes Catalyzed by Bench-Stable Alkyl Mn(I) Complexes. ACS Catal. 2019, 9, 9715–9720. 10.1021/acscatal.9b03963.

[ref24] aWeber S.; Veiros L. F.; Kirchner K. Old Concepts, New Application – Additive-Free Hydrogenation of Nitriles Catalyzed by an Air Stable Alkyl Mn(I) Complex. Adv. Synth. Catal. 2019, 361, 5412–5420. 10.1002/adsc.201901040. 31875866 PMC6916632

[ref25] Bernskoetter W. H.; Hazari N. Reversible Hydrogenation of Carbon Dioxide to Formic Acid and Methanol: Lewis Acid Enhancement of Base Metal Catalysts. Acc. Chem. Res. 2017, 50, 1049–1058. 10.1021/acs.accounts.7b00039. 28306247

[ref26] aAndersen J.-A. M.; Moss J. R. Synthesis of an Extensive Series of Manganese Pentacarbonyl Alkyl and Acyl Compounds: Carbonylation and Decarbonylation Studies on [Mn(R)(CO)_5_] and [Mn(COR)(CO)_5_]. Organometallics 1994, 13, 5013–5020. 10.1021/om00024a051.

