# Supplementary material for: Carbon Dioxide Hydrogenation to Formate Catalyzed by a Bench-Stable, Non-Pincer-Type Mn(I) Alkylcarbonyl Complex
Source: Organometallics. 2021 Apr 20;40(9):1213–20. doi: 10.1021/acs.organomet.0c00710 (PMC8155569; doi:10.1021/acs.organomet.0c00710)
Supplement: Supplementary file 1 — om0c00710_si_001.pdf [file om0c00710_si_001.pdf]

Carbon Dioxide Hydrogenation to Formate Catalyzed by a Bench-Stable, Non-Pincer Type Mn(I) Alkylcarbonyl Complex

Sylwia Kostera,[a] Stefan Weber,[b] Maurizio Peruzzini,[a] Luis F. Veiros,[c] Karl Kirchner*,[b] and Luca Gonsalvi*,[a]

[a] Consiglio Nazionale delle Ricerche (CNR), Istituto di Chimica dei Composti Organometallici (ICCOM), Via Madonna del Piano 10, 50019 Sesto Fiorentino (Firenze), Italy.

[b] Institute of Applied Synthetic Chemistry, Vienna University of Technology, Getreidemarkt 9/163-AC, A-1060 Vienna, Austria.

[c] Centro de Química Estrutural and Departamento de Engenharia Química, Instituto Superior Técnico, Universidade de Lisboa, Av Rovisco Pais, 1049-001 Lisboa, Portugal.

Supporting Information

Table of Contents

1. General Methods and Materials

2. ^{1}H NMR spectra of catalytic runs

2.1. Screening of different catalyst to base ratio, H_2/CO_2 = 1:1 (Table 1)

2.2 Screening of different H_2/CO_2 partial pressure ratios (Table 2)

2.3. Screening of the effect of Lewis acid (LA) co-catalyst (Table 3)

3. DFT calculations

1. General Methods and Materials

Complex **1** was synthesized as previously described.[1] All manipulations were carried out using standard Schlenk and glovebox techniques under nitrogen atmosphere. A H_2/CO_2 (1:1) gas mixture was purchased from Air Liquide and used as received. Different H_2/CO_2 pressure ratios were obtained by adding H_2 gas (Air Liquide) directly into the autoclave already containing a H_2/CO_2 (1:1) gas mixture at room temperature. Solvents were freshly distilled over appropriate drying agents, collected over Linde type 3Å or 4Å molecular sieves under nitrogen, and degassed with nitrogen. Deuterated solvents for NMR measurements were purchased from commercial suppliers and stored onto activated 4Å molecular sieves under nitrogen before use. The ^{1}H, $^{13}C\{^{1}H\}$, and $^{31}P\{^{1}H\}$ NMR spectra were recorded on a Bruker Avance II 300 spectrometer (operating at 300.13, 75.47, and 121.50 MHz, respectively) and a Bruker Avance II 400 spectrometer (operating at 400.13, 100.61, and 161.98 MHz, respectively) at room temperature. Peak positions are relative to tetramethylsilane and were calibrated against the residual solvent resonance (^{1}H) or the deuterated solvent multiplet (^{13}C). $^{31}P\{^{1}H\}$ NMR were referenced to 85% H_3PO_4, with the downfield shift taken as positive.

2. ^{1}H NMR spectra of catalytic runs

2.1. Screening of different catalyst to base ratio, H_2/CO_2 = 1:1 (Table 1)

Figure S1. Conditions: **1**/DBU = 1/1000, pH_2/pCO_2 (bar) = 30/30, 80 °C, 24 h (Entry 1).

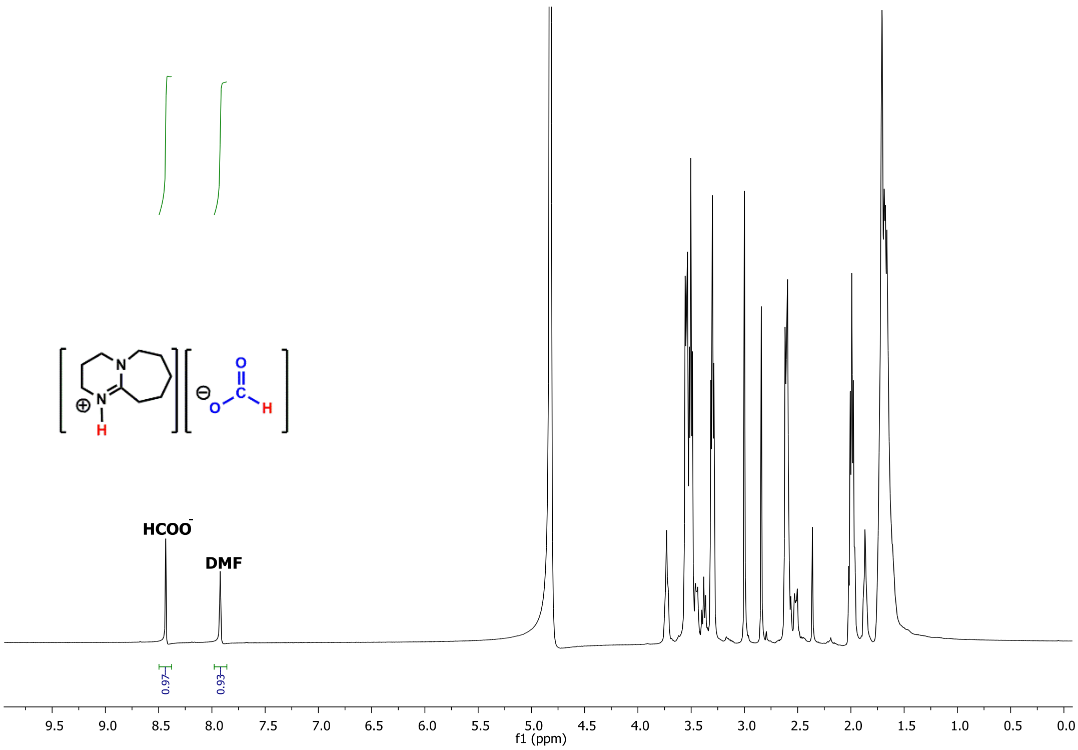

Figure S2. Conditions: **1**/DBU = 1/1000, pH_2/pCO_2 (bar) = 20/20, 80 °C, 24 h (Entry 2).

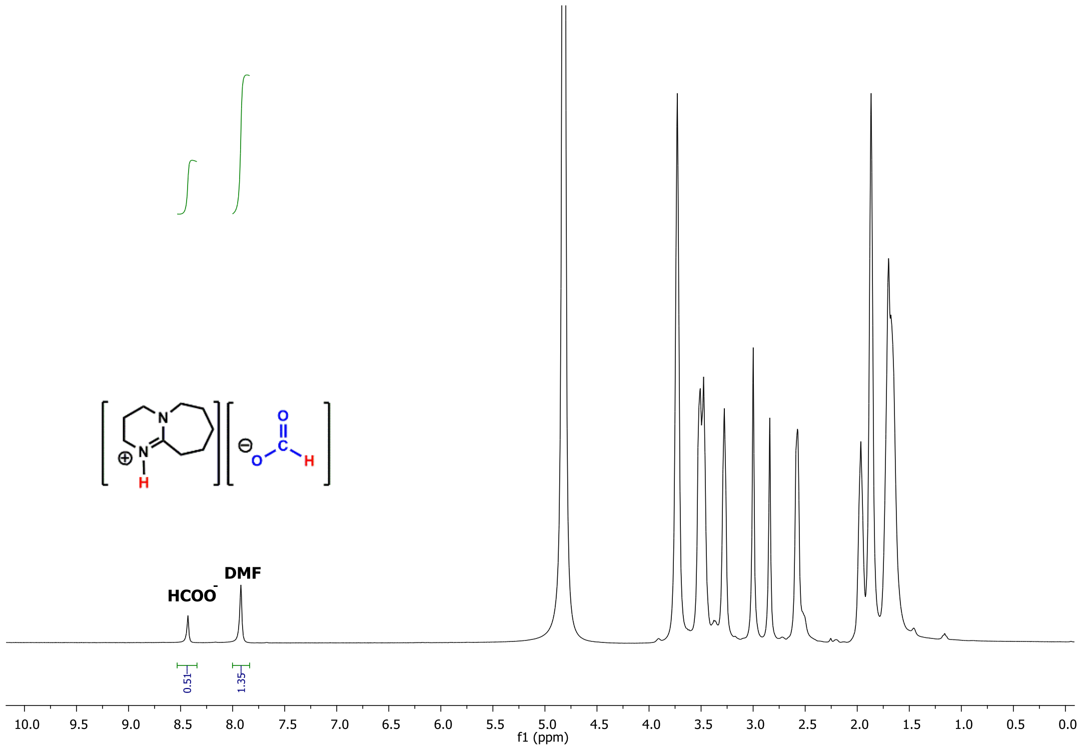

Figure S3. Conditions: **1**/DBU = 1/1000, pH_2/pCO_2 (bar) = 30/30, 80 °C, 48 h (Entry 3).

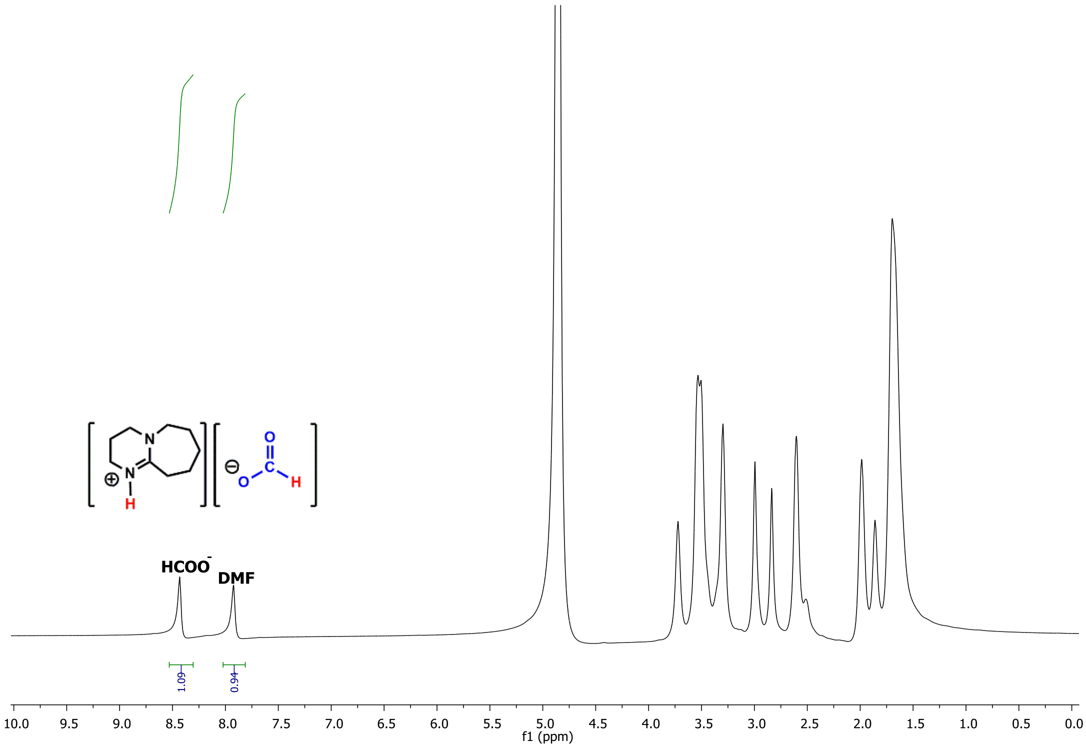

Figure S4. Conditions: **1**/DBU = 1/1000, pH_2/pCO_2 (bar) = 30/30, 80 °C, 72 h (Entry 4).

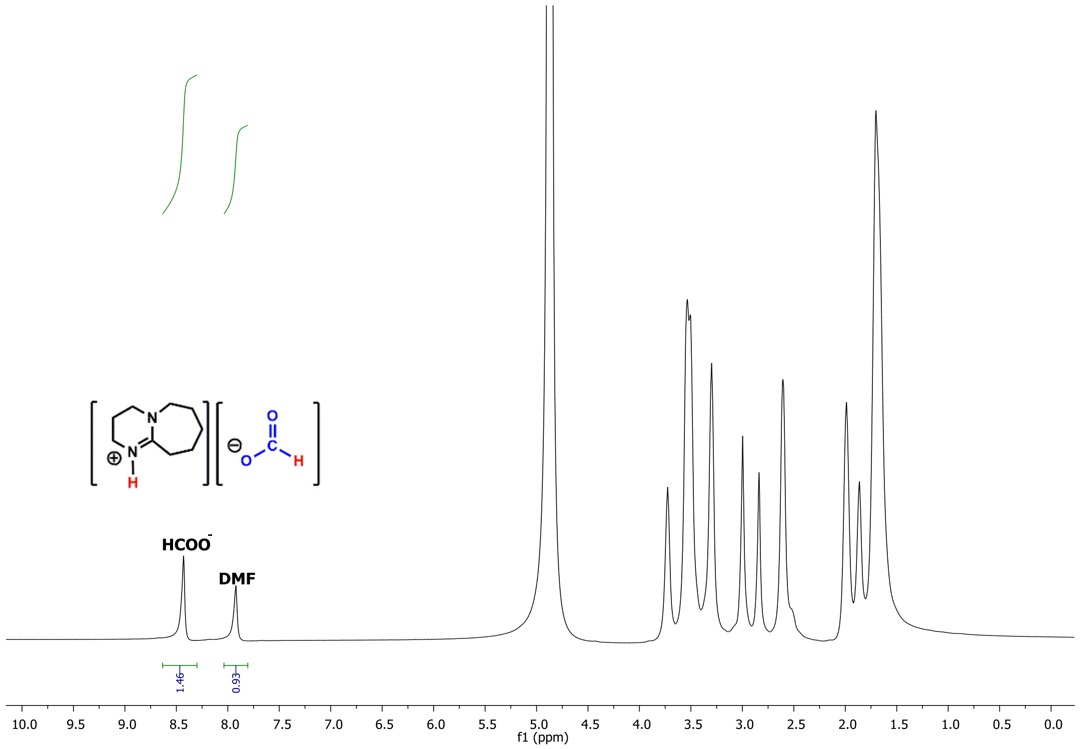

Figure S5. Conditions: **1**/DBU = 1/5000, pH_2/pCO_2 (bar) = 30/30, 80 °C, 24 h (Entry 5).

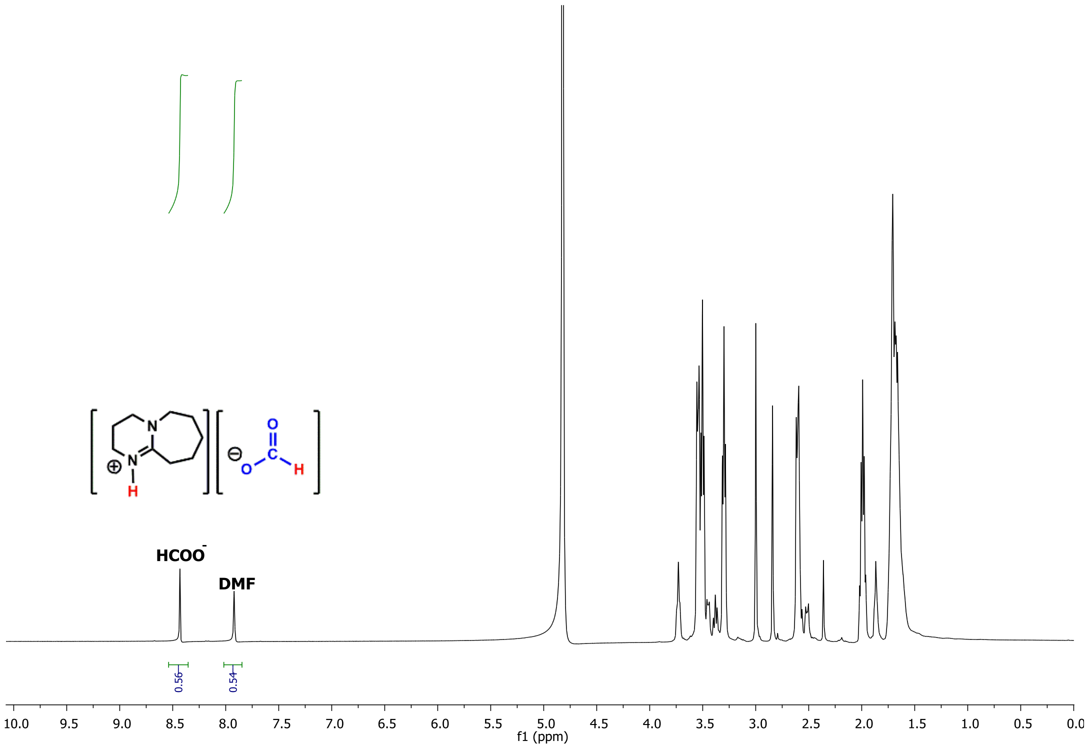

Figure S6. Conditions: **1**/DBU = 1/10000, pH_2/pCO_2 (bar) = 30/30, 80 °C, 24 h (Entry 6).

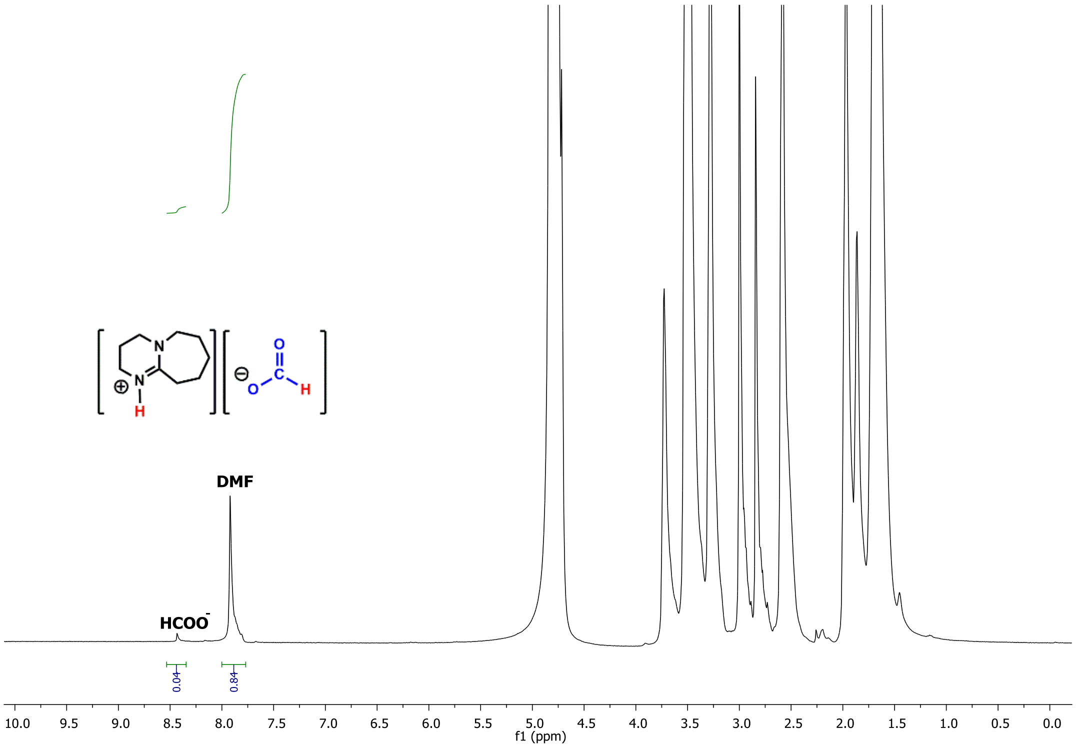

Figure S7. Conditions: **1**/DBU = 1/50000, pH_2/pCO_2 (bar) = 30/30, 80 °C, 24 h (Entry 7).

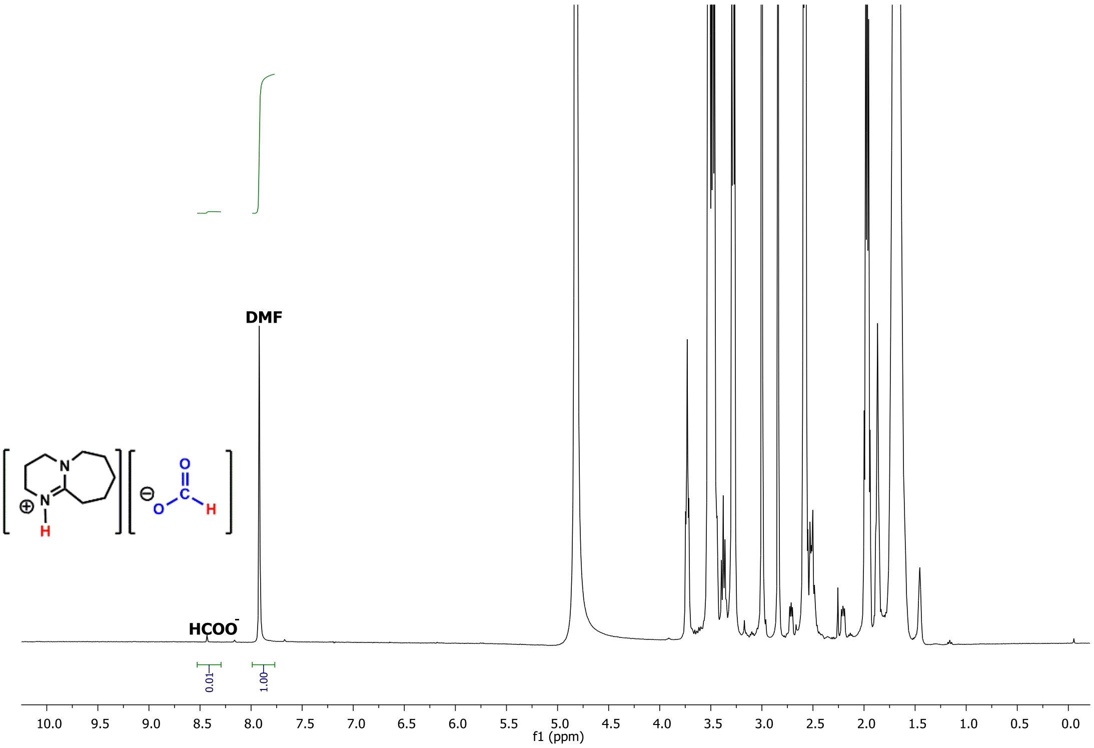

Figure S8. Conditions: **1**/DBU **=** 1/10000, pH_2/pCO_2 (bar) = 40/40, 80 °C, 24 h (Entry 8).

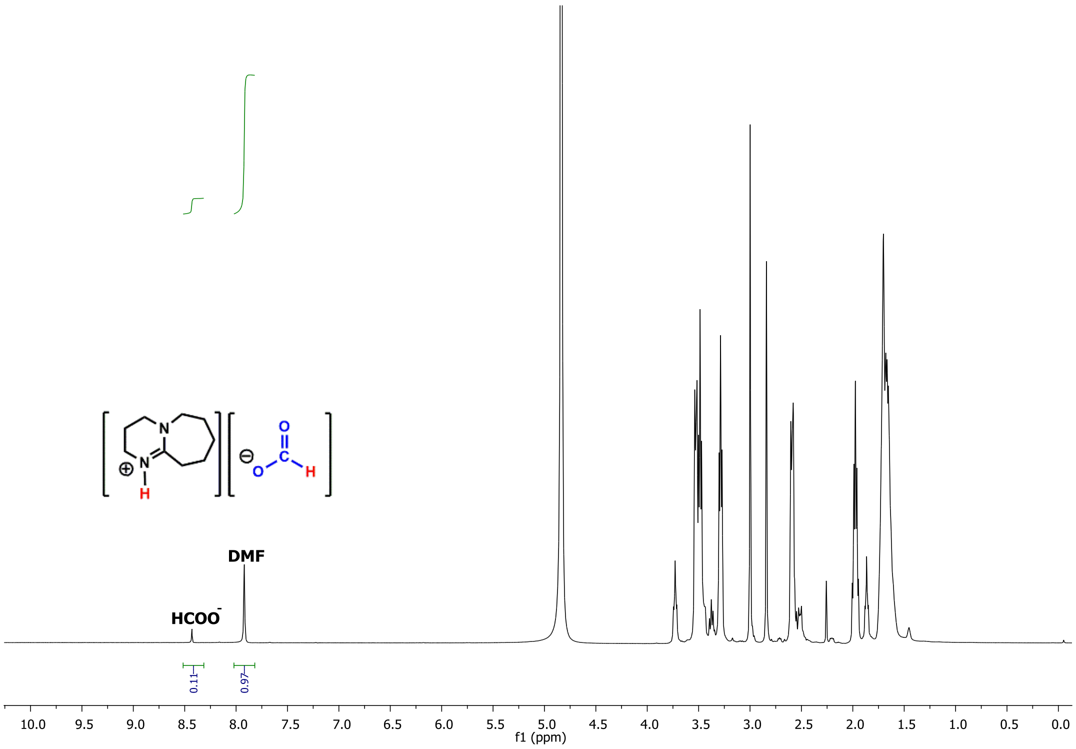

2.2. Screening of different H_2/CO_2 partial pressure ratios (Table 2)

Figure S9. Conditions: **1**/DBU **=** 1/1000, pH_2/pCO_2 (bar) = 50/25, 80 °C, 24 h (Entry 1).

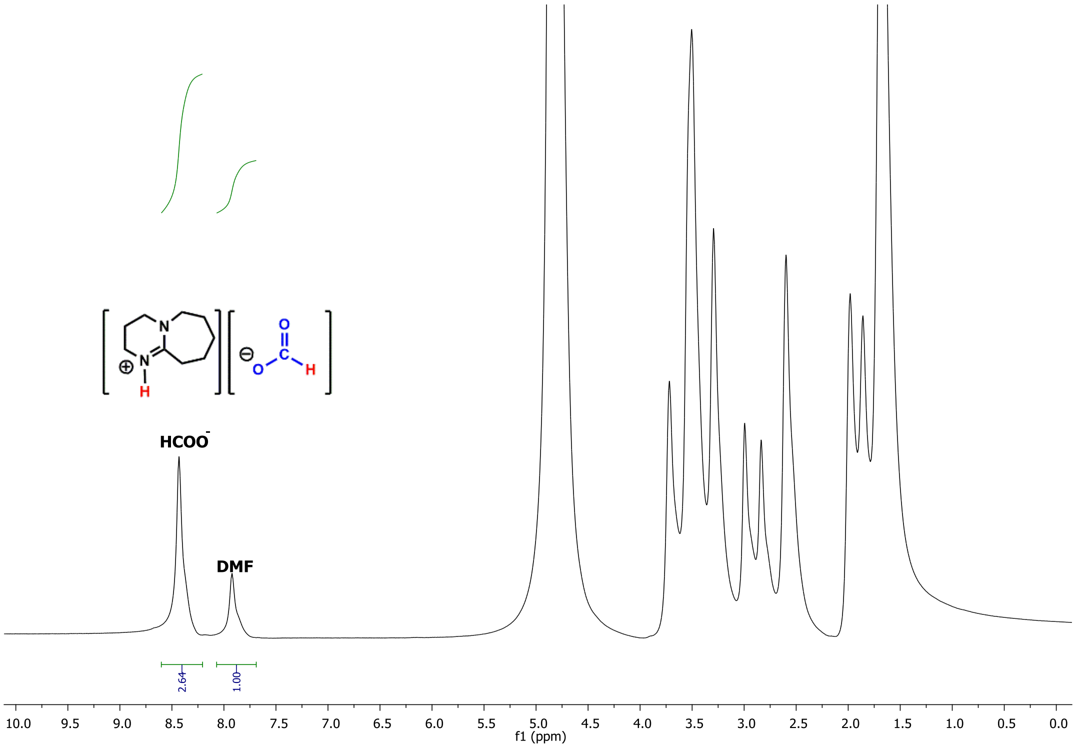

Figure S10. Conditions: **1**/DBU = 1/1000, pH_2/pCO_2 (bar) = 60/20, 80 °C, 24 h (Entry 2).

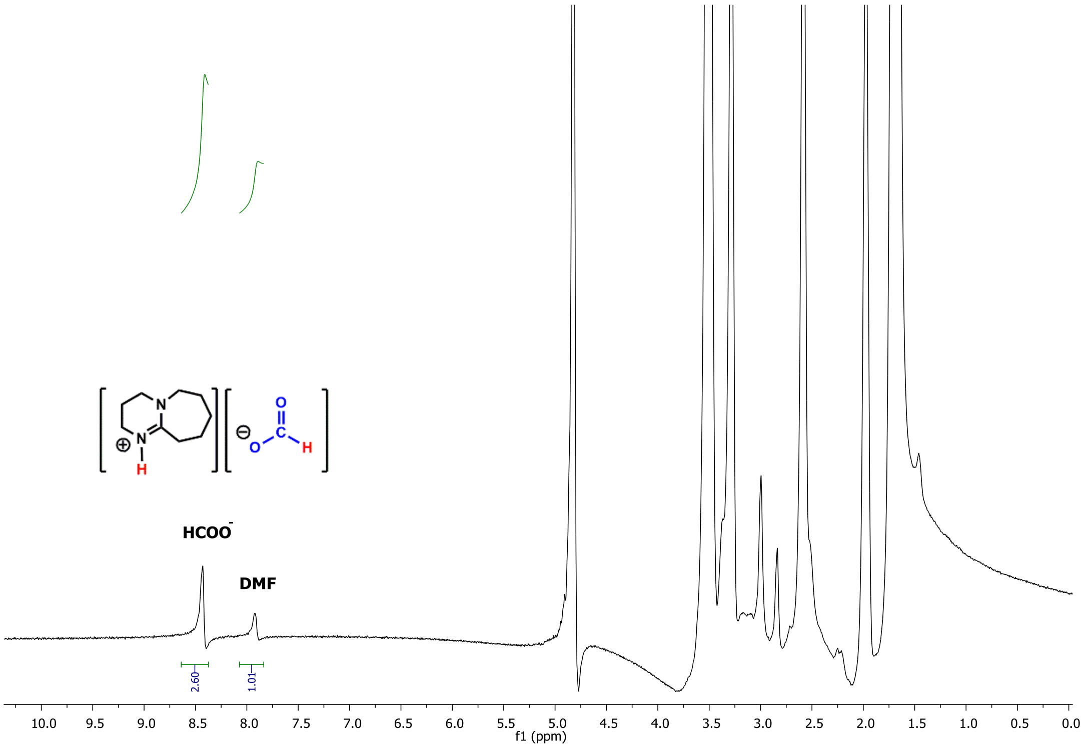

Figure S11. Conditions: **1**/DBU = 1/2000, pH_2/pCO_2 (bar) = 50/25, 80 °C, 24 h (Entry 3).

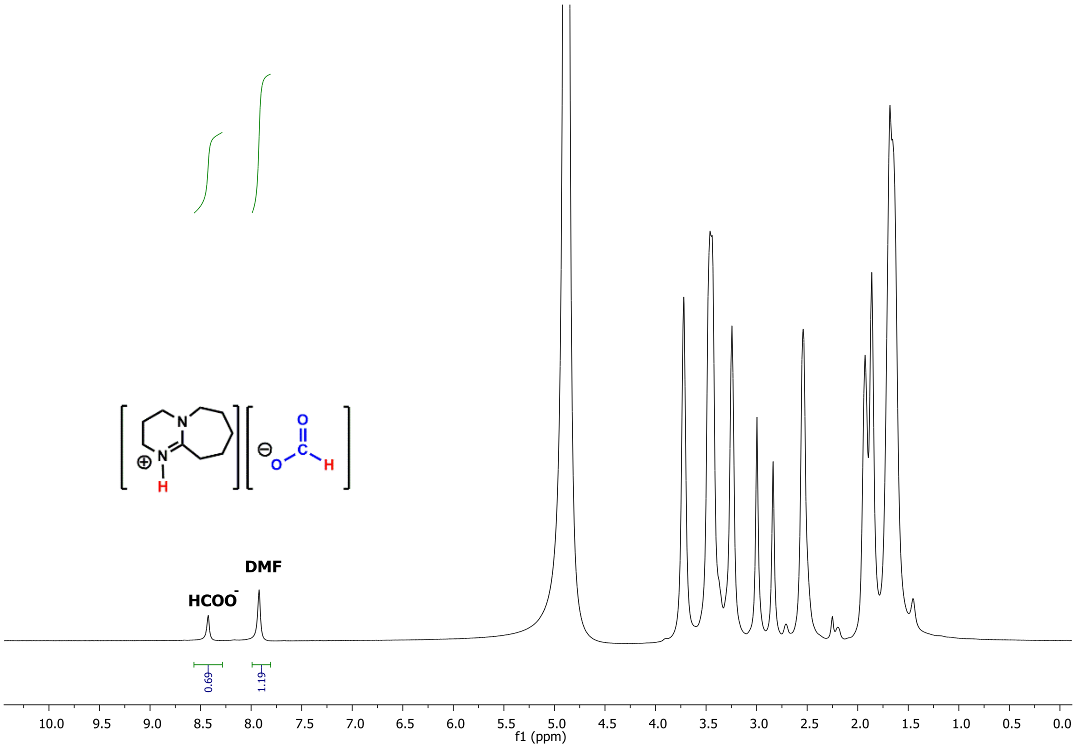

Figure S12. Conditions: **1**/DBU = 1/5000, pH_2/pCO_2 (bar) = 50/25,80 °C, 24 h (Entry 4).

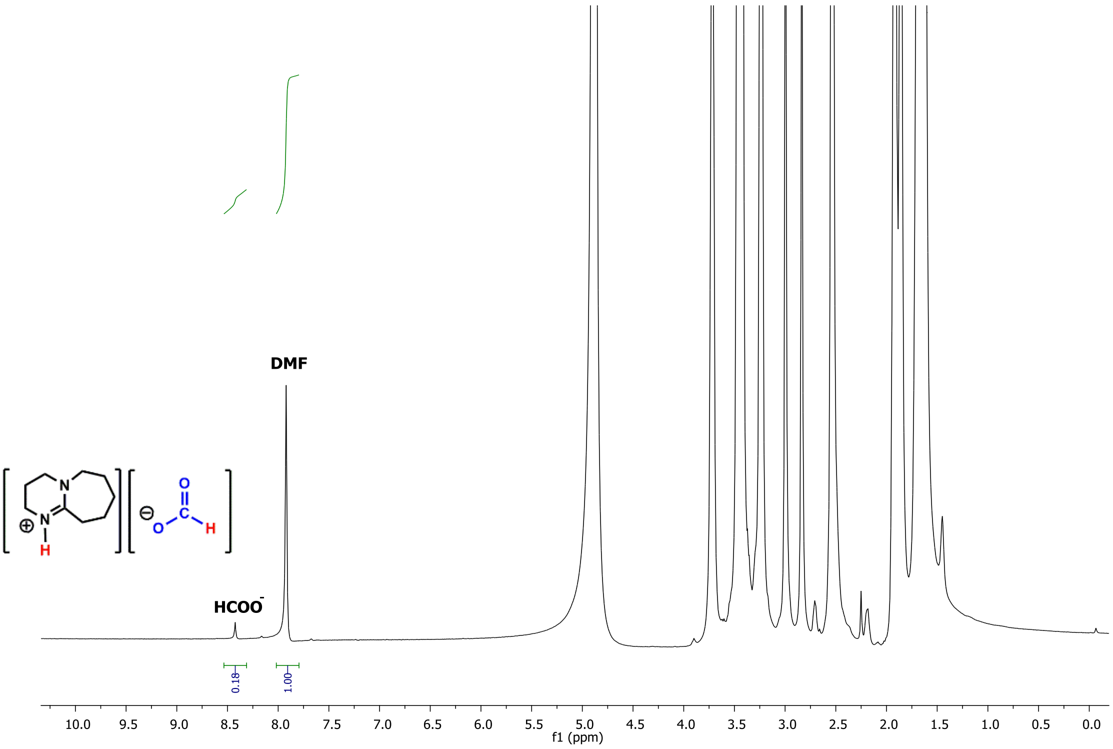

Figure S13. Conditions: **1**/DBU = 1/10000, pH_2/pCO_2 (bar) = 50/25, 80 °C, 24 h (Entry 5).

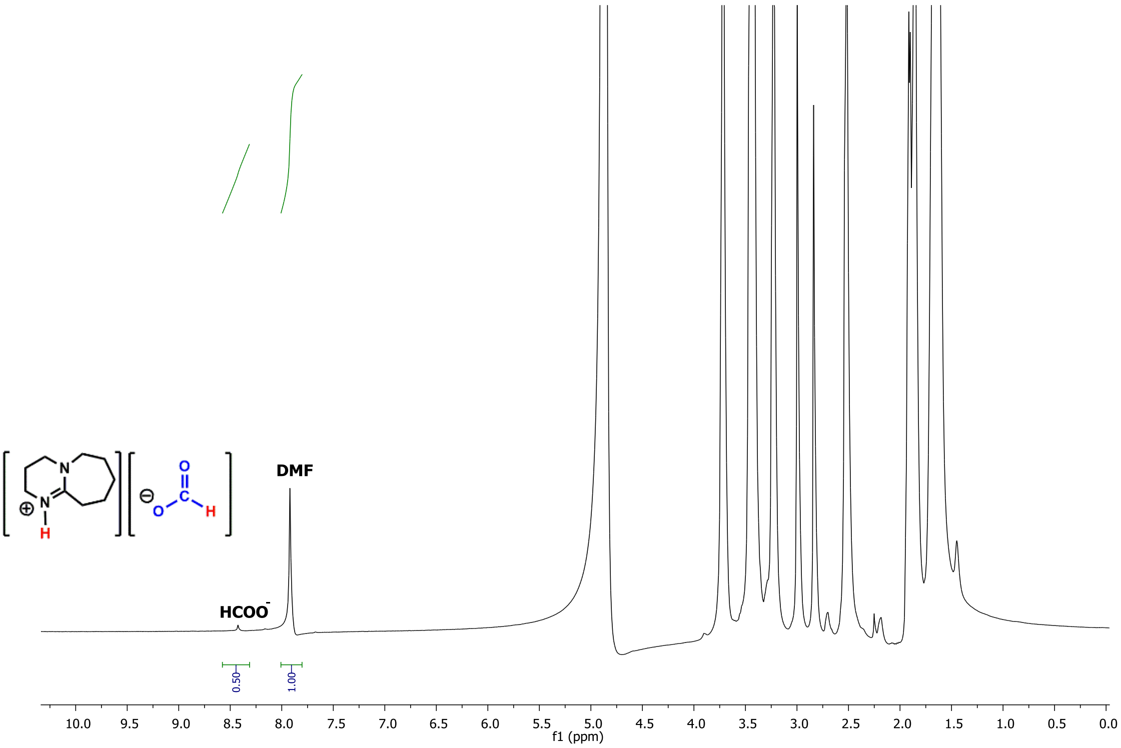

2.3. Screening of the effect of Lewis acid (LA) co-catalyst (Table 3)

Figure S14. Conditions: **1**/DBU = 1/2000, **1**/LA = 1/100, pH_2/pCO_2 (bar) = 50/25, 80 °C, 24 h (Entry 1).

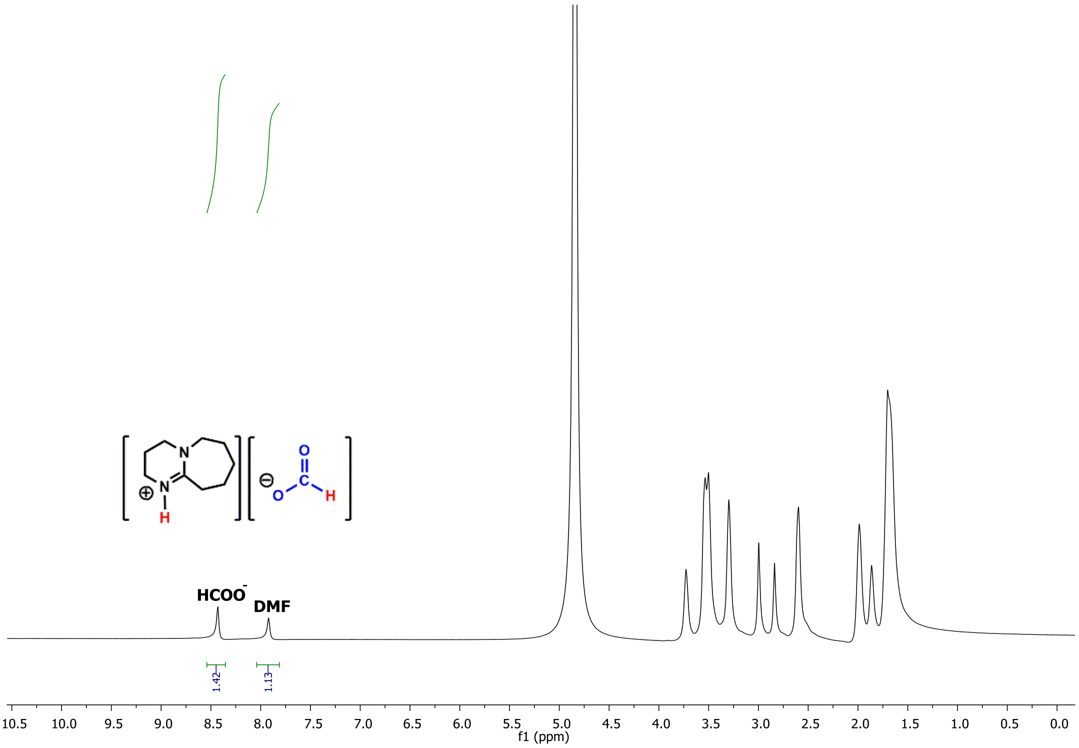

Figure S15. Conditions: **1**/DBU = 1/2000, **1**/LA = 1/100, pH_2/pCO_2 (bar) = 50/25, 80 °C, 48 h (Entry 2).

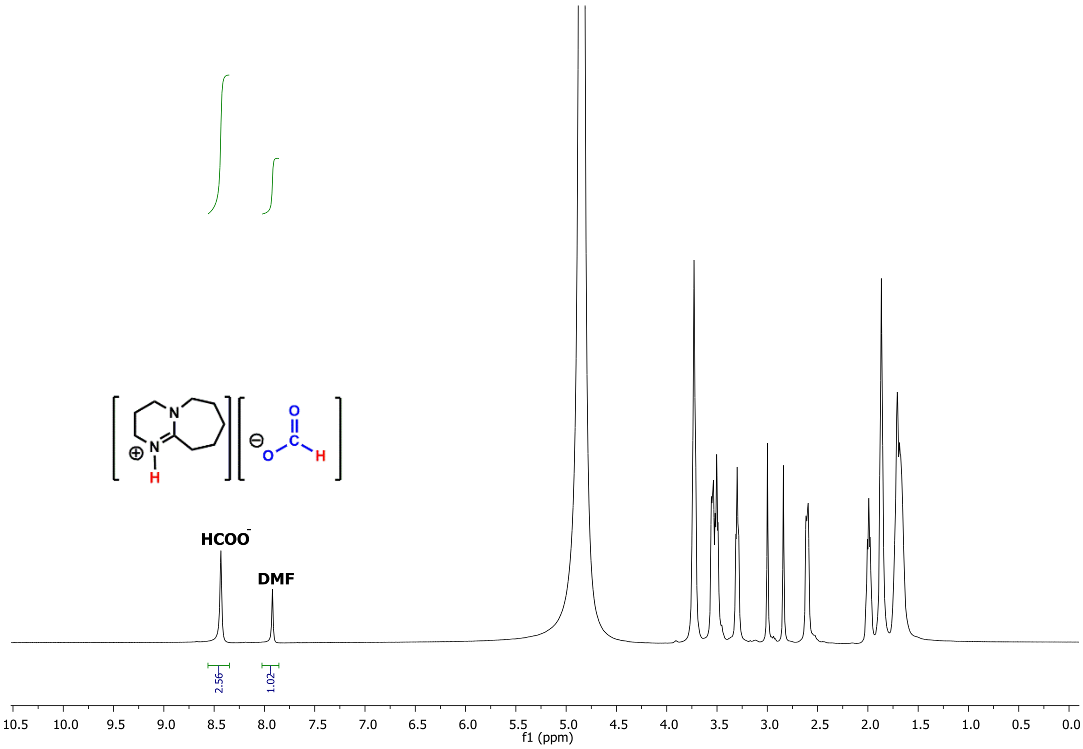

Figure S16. Conditions: **1**/DBU = 1/2000, **1**/LA = 1/100, pH_2/pCO_2 (bar) = 50/25, 100 °C, 24 h (Entry 3).

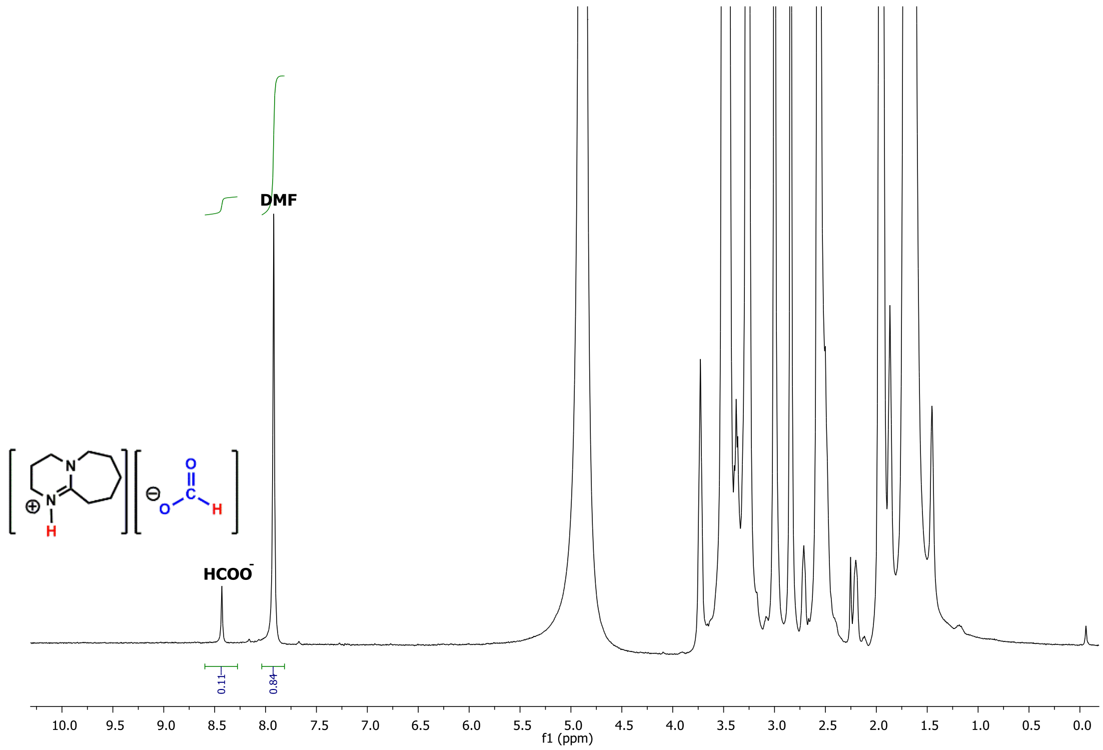

Figure S17. Conditions: **1**/DBU = 1/2000, **1**/LA = 1/200, pH_2/pCO_2 (bar) = 50/25, 80 °C, 24 h (Entry 4).

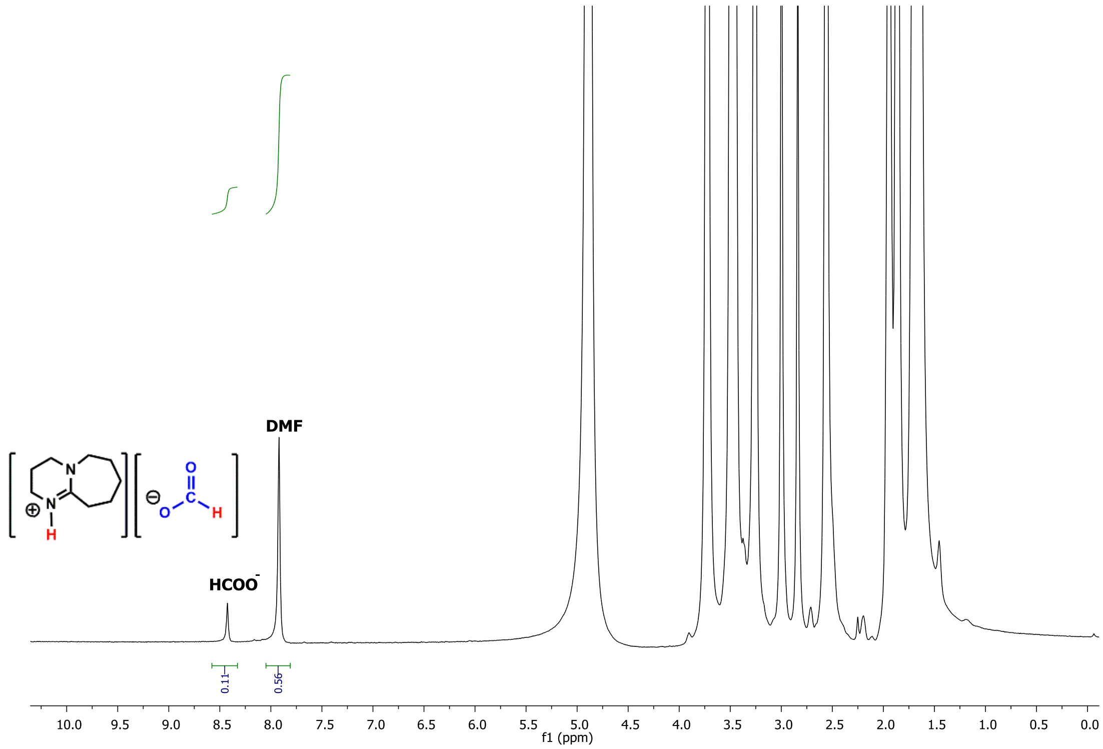

Figure S18. Conditions: **1**/DBU = 1/2000, **1**/LA = 1/50, pH_2/pCO_2 (bar) = 50/25, 80 °C, 24 h (Entry 5).

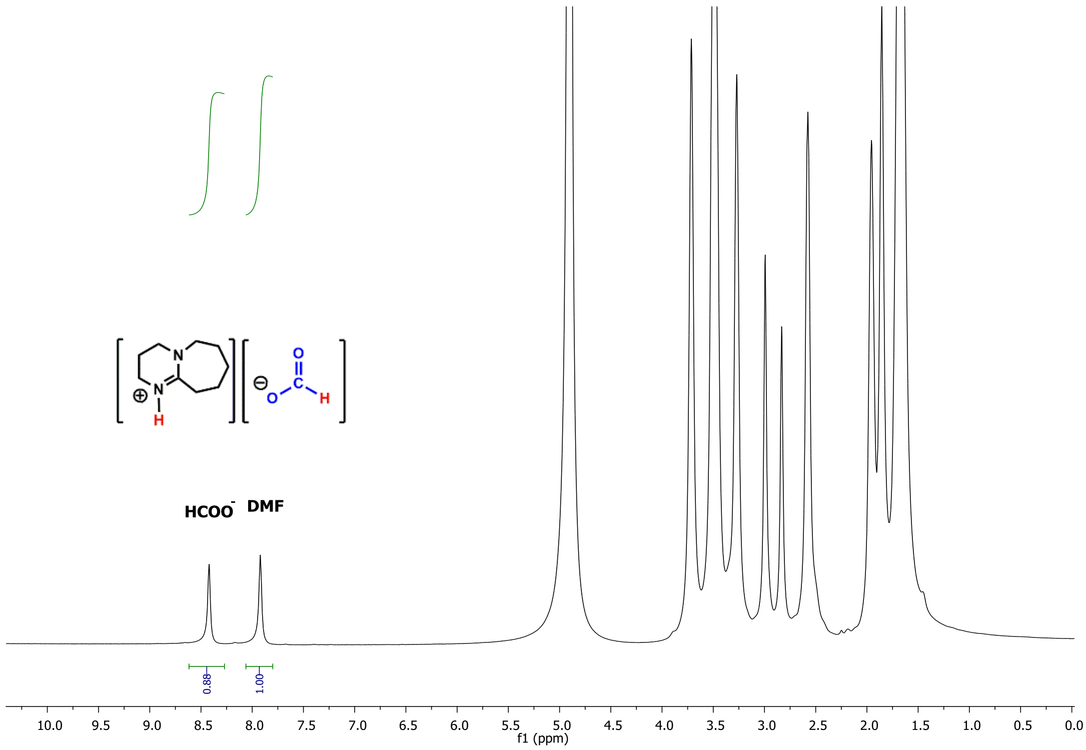

Figure S19. Conditions: **1**/DBU = 1/5000, **1**/LA = 1/250, pH_2/pCO_2 (bar) = 50/25, 80 °C, 24 h (Entry 6).

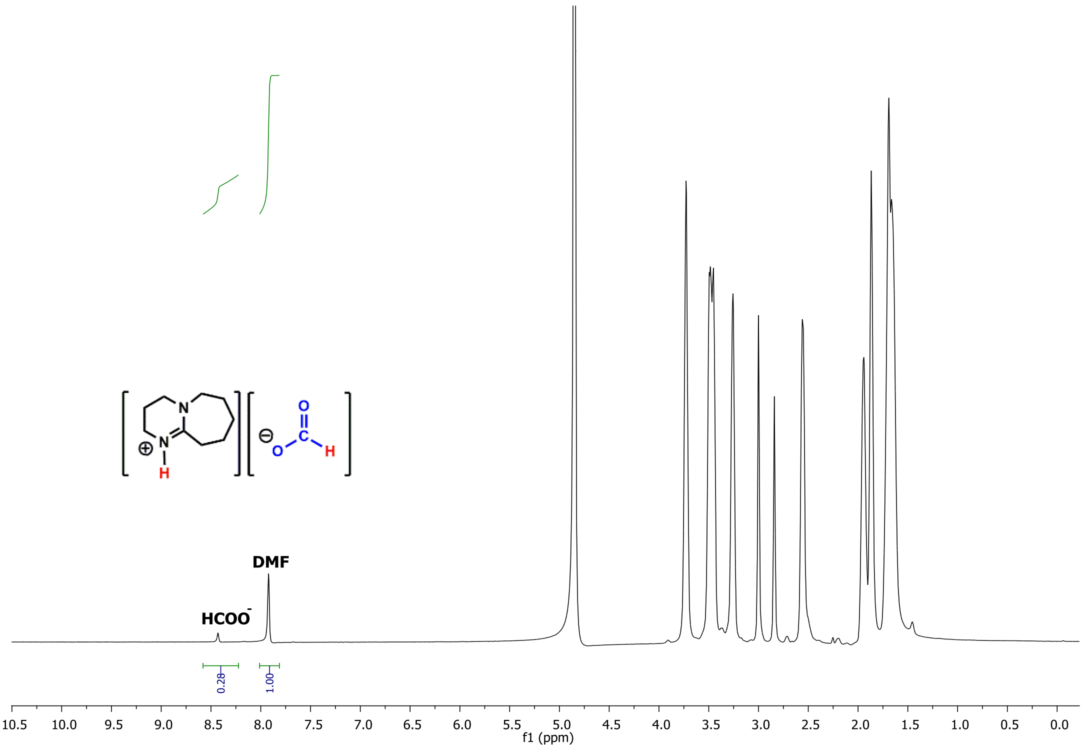

3. DFT calculations

The computational results presented have been achieved in part using the Vienna Scientific Cluster (VSC). All calculations were performed using the Gaussian 09 software package.[2] Geometry optimizations were obtained using the PBE0 functional without symmetry constraints and a basis set (b1) consisting of the Stuttgart/Dresden ECP (SDD) basis set[3] to describe the electrons of Mn, and a standard 6-31G(d,p) basis set[4] for all other atoms. The PBE0 functional uses a hybrid generalized gradient approximation (GGA), including 25% mixture of Hartree-Fock[5] exchange with DFT[6] exchange-correlation, given by Perdew, Burke and Ernzerhof functional (PBE).[7] Transition state optimizations were performed with the Synchronous Transit-Guided Quasi-Newton Method (STQN) developed by Schlegel *et al*,[8] following extensive searches of the Potential Energy Surface. Frequency calculations were performed to confirm the nature of the stationary points, yielding one imaginary frequency for the transition states and none for the minima. Each transition state was further confirmed by following its vibrational mode downhill on both sides and obtaining the minima presented on the energy profiles. The electronic energies were converted to free energy at 298.15 K and 1 atm by using zero point energy and thermal energy corrections based on structural and vibration frequency data calculated at the same level. Single point energy calculations were performed on the geometries obtained at the PBE0/b1 level using the M06 functional, and an improved basis set (b2) corresponding to same basis set for the Mn-atom and a 6-311++G(d,p) basis set[9] for the rest of the elements. The M06 functional is a hybrid meta-GGA functional developed by Truhlar and Zhao,[10] and it was shown to perform very well for the kinetics of transition metal molecules, providing a good description of weak and long range interactions.[11] Solvent effects (THF) were considered in all calculations (including the geometry optimizations) using the Polarizable Continuum Model (PCM) initially devised by Tomasi and coworkers[12] with radii and non-electrostatic terms of the SMD solvation model, developed by Truhlar *et al*.[13]

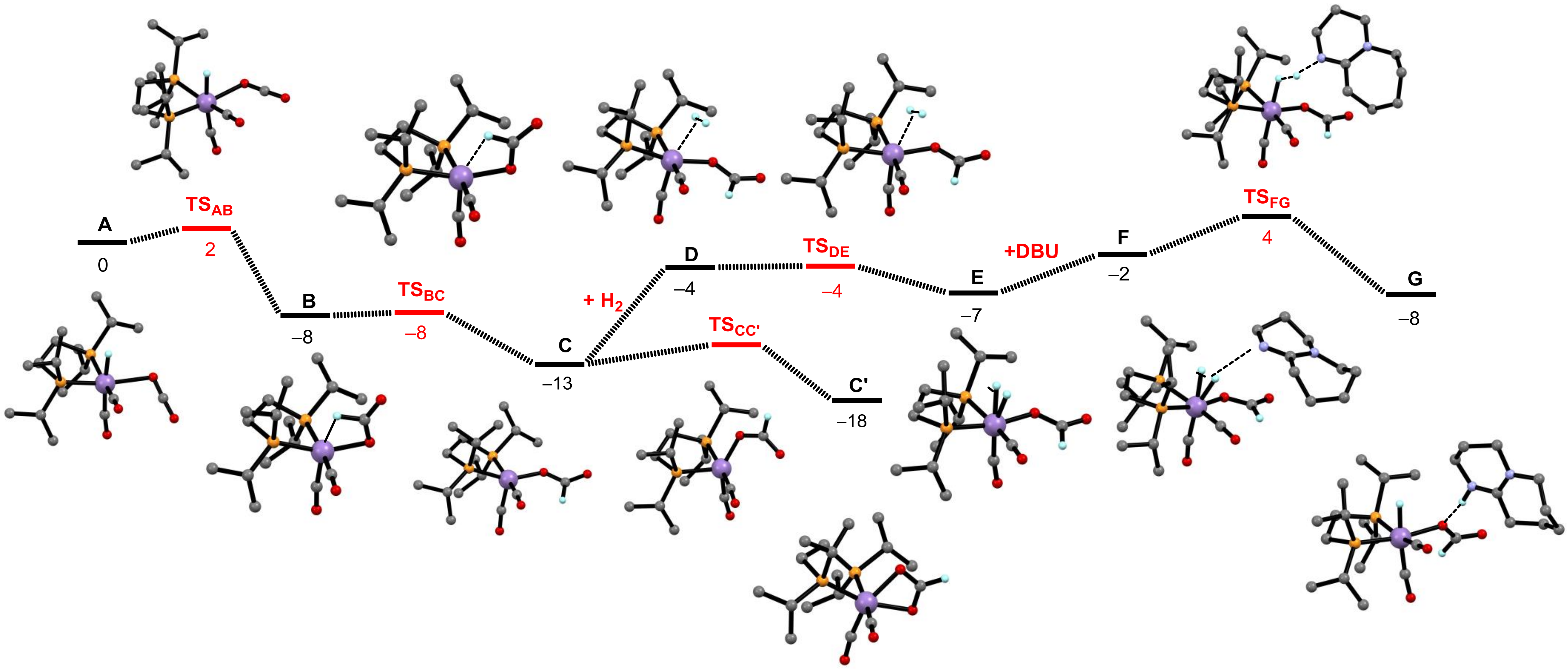

Figure S20. Inner-sphere reaction pathway for CO_2 hydrogenation from active species **A**. Free energies in kcal/mol are referred to [MnH(dippe)(κ^1-*O*-CO_2)] (**A** in the calculations).

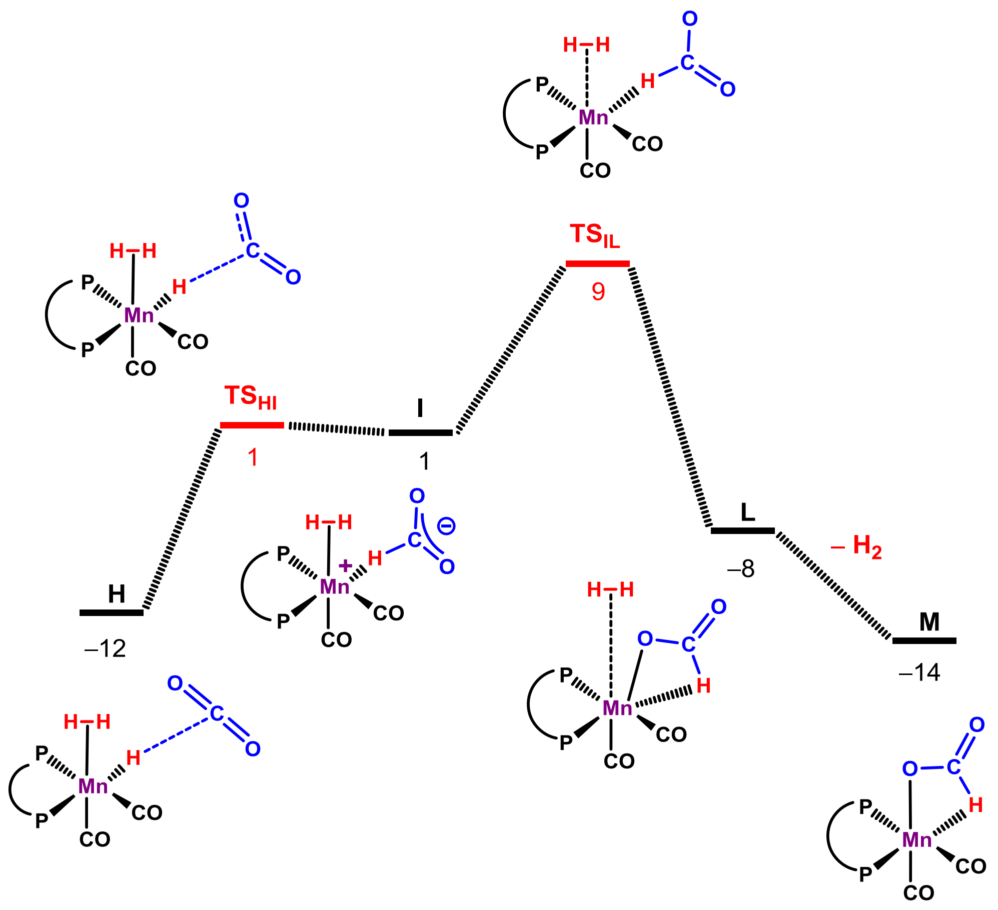

Figure S21. Alternative outer-sphere reaction pathway for CO_2 hydrogenation starting from active species **H**. Free energies in kcal/mol are referred to [MnH(dippe)(κ^1-*O*-CO_2)] (**A** in the calculations, see Figure S20).

REFERENCES

(1) Weber, S.; Stöger, B.; Veiros, L. F.; Kirchner, K. Rethinking Basic Concepts - Hydrogenation of Alkenes Catalyzed by Bench-Stable Alkyl Mn(I) Complexes, *ACS Catal.* **2019**, *9*, 9715−9720.

(2) Gaussian 09, Revision **A.01**, Frisch, M. J.; Trucks, G. W.; Schlegel, H. B.; Scuseria, G. E.; Robb, M. A.; Cheeseman, J. R.; Scalmani, G.; Barone, V.; Mennucci, B.; Petersson, G. A.; Nakatsuji, H.; Caricato, M.; Li, X.; Hratchian, H. P.; Izmaylov, A. F.; Bloino, J.; Zheng, G.; Sonnenberg, J. L.; Hada, M.; Ehara, M.; Toyota, K.; Fukuda, R.; Hasegawa, J.; Ishida, M.; Nakajima, T.; Honda, Y.; Kitao, O.; Nakai, H.; Vreven, T.; Montgomery, Jr., J. A.; Peralta, J. E.; Ogliaro, F.; Bearpark, M.; Heyd, J. J.; Brothers, E.; Kudin, K. N.; Staroverov, V. N.; Kobayashi, R.; Normand, J.; Raghavachari, K.; Rendell, A.; Burant, J. C.; Iyengar, S. S.; Tomasi, J.; Cossi, M.; Rega, N.; Millam, J. M.; Klene, M.; Knox, J. E.; Cross, J. B.; Bakken, V.; Adamo, C.; Jaramillo, J.; Gomperts, R.; Stratmann, R. E.; Yazyev, O.; Austin, A. J.; Cammi, R.; Pomelli, C.; Ochterski, J. W.; Martin, R. L.; Morokuma, K.; Zakrzewski, V. G.; Voth, G. A.; Salvador, P.; Dannenberg, J. J.; Dapprich, S.; Daniels, A. D.; Farkas, Ö.; Foresman, J. B.; Ortiz, J. V.; Cioslowski, J.; Fox, D. J. Gaussian, Inc., Wallingford CT, 2009.

(3) a) Haeusermann, U.; Dolg, M.; Stoll, H.; Preuss, H.; Schwerdtfeger, P.; Pitzer, R. M. Accuracy of energy-adjusted quasirelativistic ab initio pseudopotentials *Mol. Phys.* **1993**, *78*, 1211-1224. b) Kuechle, W.; Dolg, M.; Stoll, H.; Preuss, H. Energy- adjusted pseudopotentials for the actinides. Parameter sets and test calculations for thorium and thorium monoxide *J. Chem. Phys.* **1994**, *100*, 7535-7542. c) Leininger, T.; Nicklass, A.; Stoll, H.; Dolg, M.; Schwerdtfeger, P. The accuracy of the pseudopotential approximation. II. A comparison of various core sizes for indium pseudopotentials in calculations for spectroscopic constants of InH, InF, and InCl *J. Chem. Phys.* **1996**, *105*, 1052-1059.

(4) a) Ditchfield, R.; Hehre, W. J.; Pople, J. A. Self-Consistent Molecular-Orbital Methods. IX. An Extended Gaussian-Type Basis for Molecular-Orbital Studies of Organic Molecules *J. Chem. Phys.* **1971**, *54*, 724-728. b) Hehre, W. J.; Ditchfield, R.; Pople, J. A. Self-Consistent Molecular Orbital Methods. 12. Further extensions of Gaussian-type basis sets for use in molecular-orbital studies of organic-molecules *J. Chem. Phys.* **1972**, *56*, 2257-2261. c) Hariharan, P. C.; Pople, J. A. Accuracy of AH equilibrium geometries by single determinant molecular-orbital theory *Mol. Phys.* **1974**, *27*, 209-214. d) Gordon, M. S. The isomers of silacyclopropane *Chem. Phys. Lett.* **1980**, *76*, 163-168. e) Hariharan, P. C.; Pople, J. A. Influence of polarization functions on molecular-orbital hydrogenation energies *Theor. Chim. Acta* **1973**, *28*, 213-222.

(5) Hehre, W. J., Radom, L., Schleyer, P. v.R. & Pople, J. A. *Ab Initio Molecular Orbital Theory*, John Wiley & Sons, NY, 1986.

(6) Parr, R. G. & Yang, W. *Density Functional Theory of Atoms and Molecules*; Oxford University Press: New York, 1989.

(7) a) Perdew, J. P.; Burke, K.; Ernzerhof, M. Generalized Gradient Approximation Made Simple *Phys. Rev. Lett.* **1996**, *77*, 3865-3868; b) Perdew, J. P.; Burke, K.; Ernzerhof, M. Generalized Gradient Approximation Made Simple *Phys. Rev. Lett.* **1997**, *78*, 1396-1396. c) Perdew, J. P. Density-functional approximation for the correlation energy of the inhomogeneous electron gas *Phys. Rev. B* **1986**, *33*, 8822-8824.

(8) a) Peng, C.; Ayala, P. Y.; Schlegel, H. B.; Frisch, M. J. Using redundant internal coordinates to optimize equilibrium geometries and transition states *J. Comp. Chem.* **1996**, *17*, 49-56. b) Peng, C.; Schlegel, H. B. Combining Synchronous Transit and Quasi-Newton Methods for Finding Transition States *Israel J. Chem.* **1993**, *33*, 449-454.

(9) a) McClean, A. D.; Chandler, G. S. Contracted Gaussian basis sets for molecular calculations. I. Second row atoms, *Z*=11-18 *J. Chem. Phys.* **1980**, *72*, 5639-5648. b) Krishnan, R.; Binkley, J. S.; Seeger, R.; Pople, J. A. Self-consistent molecular orbital methods. XX. A basis set for correlated wave functions *J. Chem. Phys.* **1980**, *72*, 650-654. c) Wachters, A. J. H. Gaussian Basis Set for Molecular Wavefunctions Containing Third-Row Atoms *J. Chem. Phys.* **1970**, *52*, 1033-1036. d) Hay, P. J. Gaussian basis sets for molecular calculations - representation of 3D orbitals in transition-metal atoms *J. Chem. Phys.* **1977**, *66*, 4377-4384. e) Raghavachari, K.; Trucks, G. W. Highly correlated systems: Excitation energies of first row transition metals Sc-Cu *J. Chem. Phys.* **1989**, *91*, 1062-1065. f) Binning Jr., R. C.; Curtiss, L. A. Compact contracted basis-sets for 3rd-row atoms - Ga-Kr *J. Comp. Chem.* **1990**, *11*, 1206-1216. g) McGrath, M. P.; Radom, L. Extension of Gaussian-1 (G1) theory to bromine-containing molecules *J. Chem. Phys.* **1991**, *94*, 511-516. h) Curtiss, L. A.; McGrath, M. P.; Blaudeau, J.-P.; Davis, N. E.; Binning Jr., R. C.;

Radom, L. Extension of Gaussian-2 theory to molecules containing third-row atoms Ga-Kr *J. Chem. Phys.*, **1995**, *103*, 6104-6113. i) Clark, T.; Chandrasekhar, J.; Spitznagel, G. W.; Schleyer, P. v. R. Efficient diffuse function-augmented basis-sets for anion calculations. 3. The 3-21+G basis set for 1st-row elements, Li-F *J. Comp. Chem.* **1983**, *4*, 294-301. j) Frisch, M. J.; Pople, J. A.; Binkley, J. S. Self-Consistent Molecular Orbital Methods. 25. Supplementary Functions for Gaussian Basis Sets *J. Chem. Phys.* **1984**, *80*, 3265-3269.

(10) Zhao, Y.; Truhlar, D. G. The M06 suite of density functionals for main group thermochemistry, thermochemical kinetics, noncovalent interactions, excited states, and transition elements: two new functionals and systematic testing of four M06-class functionals and 12 other functionals *Theor. Chem. Acc.*, **2008**, *120*, 215-241.

(11) a) Zhao, Y.; Truhlar, D. G. Density Functionals with Broad Applicability in Chemistry *Acc. Chem. Res.* **2008**, *41*, 157-167. b) Zhao, Y.; Truhlar, D. G. Applications and validations of the Minnesota density functionals *Chem. Phys. Lett.* **2011**, *502*, 1-13.

(12) a) Cancès, M. T.; Mennucci, B.; Tomasi, J. A new integral equation formalism for the polarizable continuum model: Theoretical background and applications to isotropic and anistropic dielectrics *J. Chem. Phys.* **1997**, *107*, 3032-3041. b) Cossi, M.; Barone, V.; Mennucci, B.; Tomasi, J. Ab initio study of ionic solutions by a polarizable continuum dielectric model *Chem. Phys. Lett.* **1998**, *286*, 253-260. c) Mennucci, B.; Tomasi, J. Continuum solvation models: A new approach to the problem of solute's charge distribution and cavity boundaries *J. Chem. Phys.* **1997**, *106*, 5151-5158. d) Tomasi, J.; Mennucci, B.; Cammi, R. Quantum mechanical continuum solvation models *Chem. Rev*. **2005**, *105*, 2999-3094.

(13) Marenich, A. V.; Cramer, C. J.; Truhlar, D. G. Universal solvation model based on solute electron density and a continuum model of the solvent defined by the bulk dielectric constant and atomic surface tensions *J. Phys. Chem. B*, **2009**, *113*, 6378-6396.
